# Genome-Wide Analysis of Left Ventricular Image-Derived Phenotypes Identifies Fourteen Loci Associated With Cardiac Morphogenesis and Heart Failure Development

**DOI:** 10.1161/CIRCULATIONAHA.119.041161

**Published:** 2019-09-25

**Authors:** Nay Aung, Jose D. Vargas, Chaojie Yang, Claudia P. Cabrera, Helen R. Warren, Kenneth Fung, Evan Tzanis, Michael R. Barnes, Jerome I. Rotter, Kent D. Taylor, Ani W. Manichaikul, Joao A.C. Lima, David A. Bluemke, Stefan K. Piechnik, Stefan Neubauer, Patricia B. Munroe, Steffen E. Petersen

**Affiliations:** 1 Centre for Translational Bioinformatics (C.P.C., E.T., M.R.B.), Queen Mary University of London, United Kingdom.; 2 William Harvey Research Institute, Barts and The London School of Medicine and Dentistry (N.A., H.R.W., K.F., P.B.M., S.E.P.), Queen Mary University of London, United Kingdom.; 3 National Institute for Health Research, Barts Cardiovascular Biomedical Research Centre (N.A., H.R.W., K.F., P.B.M., S.E.P.), Queen Mary University of London, United Kingdom.; 4 Barts Heart Centre, St Bartholomew’s Hospital, Barts Health National Health Service Trust, West Smithfield, London, United Kingdom (N.A., K.F., S.E.P.).; 5 Medstar Heart and Vascular Institute, Medstar Georgetown University Hospital, Washington, DC (J.D.V.).; 6 Center for Public Health Genomics, University of Virginia, Charlottesville (C.Y., A.W.M.).; 7 The Institute for Translational Genomics and Population Sciences, Division of Genomics Outcomes, Department of Pediatrics, Los Angeles Biomedical Research Institute at Harbor-University of California, Los Angeles, Medical Center, Torrance, CA (J.I.R., K.D.T.).; 8 Division of Cardiology, Johns Hopkins University, Baltimore, MD (J.AC.L.).; 9 Department of Radiology, University of Wisconsin, Madison (D.A.B.).; 10 Division of Cardiovascular Medicine, Radcliffe Department of Medicine, University of Oxford, United Kingdom (S.K.P., S.N.)

**Keywords:** genome-wide association study, heart failure, left ventricle

## Abstract

The genetic basis of left ventricular (LV) image-derived phenotypes, which play a vital role in the diagnosis, management, and risk stratification of cardiovascular diseases, is unclear at present.

**Methods:**

The LV parameters were measured from the cardiovascular magnetic resonance studies of the UK Biobank. Genotyping was done using Affymetrix arrays, augmented by imputation. We performed genome-wide association studies of 6 LV traits—LV end-diastolic volume, LV end-systolic volume, LV stroke volume, LV ejection fraction, LV mass, and LV mass to end-diastolic volume ratio. The replication analysis was performed in the MESA study (Multi-Ethnic Study of Atherosclerosis). We identified the candidate genes at genome-wide significant loci based on the evidence from extensive bioinformatic analyses. Polygenic risk scores were constructed from the summary statistics of LV genome-wide association studies to predict the heart failure events.

**Results:**

The study comprised 16 923 European UK Biobank participants (mean age 62.5 years; 45.8% men) without prevalent myocardial infarction or heart failure. We discovered 14 genome-wide significant loci (3 loci each for LV end-diastolic volume, LV end-systolic volume, and LV mass to end-diastolic volume ratio; 4 loci for LV ejection fraction, and 1 locus for LV mass) at a stringent *P*<1×10^−8^. Three loci were replicated at Bonferroni significance and 7 loci at nominal significance (*P*<0.05 with concordant direction of effect) in the MESA study (n=4383). Follow-up bioinformatic analyses identified 28 candidate genes that were enriched in the cardiac developmental pathways and regulation of the LV contractile mechanism. Eight genes (*TTN, BAG3, GRK5, HSPB7, MTSS1, ALPK3, NMB*, and *MMP11*) supported by at least 2 independent lines of in silico evidence were implicated in the cardiac morphogenesis and heart failure development. The polygenic risk scores of LV phenotypes were predictive of heart failure in a holdout UK Biobank sample of 3106 cases and 224 134 controls (odds ratio 1.41, 95% CI 1.26 – 1.58, for the top quintile versus the bottom quintile of the LV end-systolic volume risk score).

**Conclusions:**

We report 14 genetic loci and indicate several candidate genes that not only enhance our understanding of the genetic architecture of prognostically important LV phenotypes but also shed light on potential novel therapeutic targets for LV remodeling.

## Abstract 2

Supplemental Digital Content is available in the text.

**Clinical Perspective**

**What Is New?**

- Prognostically important left ventricular imaging phenotypes are highly heritable (~22% to 39%).
- A total of 14 genetic susceptibility loci (8 of which are unique) enriched in the cardiac developmental pathways and regulation of contractile mechanism are discovered in the largest genome-wide association study of cardiovascular magnetic resonance-derived left ventricular phenotypes.
- The polygenic risk scores of left ventricular phenotypes are predictive of heart failure events independently of clinical risk factors.

**What Are the Clinical Implications?**

- The findings from this study not only enhance our understanding of the genetic basis of prognostically important left ventricular phenotypes in the general population but also underscore the intricate genetic relationship between these endophenotypes and the pathogenesis of the heart failure syndrome.
- The prioritized genes in the genome-wide significant loci should be followed up in the functional studies to aid the development of potential novel therapies for heart failure.
- The polygenic risk scores of left ventricular phenotypes may have a role in personalized risk stratification pending further validation of clinical robustness in future studies.

Heart failure is a clinically heterogeneous condition associated with a substantial mortality, morbidity, and economic burden to the society.^1^ Globally, both incidence and prevalence of heart failure are increasing due to improved survival from other contributory cardiovascular diseases in an aging population. The diagnosis and treatment of heart failure is in part based on left ventricular (LV) functional and structural parameters derived from cardiac imaging. Although the impact of modifiable risk factors on LV structure and function is well established, our current understanding of the genetic component of these imaging phenotypes is limited.

Previous large-scale genetic association studies of LV imaging phenotypes^2,3^ were hampered by the lack of a standardized measurement protocol in the phenotyping process and reliance on 2-dimensional echocardiography (ECHO) with inherent dependency on geometric assumptions and adequate acoustic window. These shortcomings can be overcome by using the individual-level data from a single large study such as the UK Biobank, which also provides accurate and reproducible imaging phenotypes from cardiovascular magnetic resonance (CMR) imaging, considered to be a reference standard for the assessment of cardiac morphology.^4^

Although multiple indices of LV structure and function can be measured from CMR images, 5 parameters, LV mass (LVM), LV end-diastolic volume (LVEDV), LV end-systolic volume (LVESV), LV stroke volume (LVSV), and LV ejection fraction (LVEF), are frequently used in clinical practice and carry prognostic information.^5,6^ In addition, LV mass to end-diastolic volume ratio (LVMVR) represents geometric remodeling of the left ventricle, and an elevated LVMVR reflects concentric remodeling or hypertrophy associated with adverse outcomes.^7^ Systematic genome-wide scanning for loci associated with the LV image-derived measurements is a vital first step, which will advance our understanding of their genetic basis in a general population and may inform novel diagnostic and targeted therapeutic opportunities. In this study, we conducted genome-wide association studies (GWASs) to identify the genetic loci for 6 clinically relevant CMR-derived LV imaging phenotypes.

## Methods

### Data Access

The data including GWAS summary statistics, analytic methods, and study materials will be returned to the UK Biobank. The UK Biobank will make these data available to all bona fide researchers for all types of health-related research that is in the public interest, without preferential or exclusive access for any person. Please see the UK Biobank’s website for the detailed access procedure (http://www.ukbiobank.ac.uk/register-apply/).

### UK Biobank

The UK Biobank is a large population-based prospective cohort study of a half million people aged between 40 and 69 years at the time of initial recruitment between 2006 and 2010. It has collected a wealth of information on health and lifestyle data, physical measurements, biological samples, genotype, and cardiac phenotypes derived from CMR. The study protocol has been described in detail previously.^8^ The study complies with the Declaration of Helsinki and was approved by our institutional review body. All participants provided informed written consent.

### Genetic Data

Genotypes directly called by 2 closely related, purpose-built arrays known as UK Biobank Axiom Array (825 927 markers) and UK BiLEVE Axiom Array (807 411 markers) were imputed to ~92 million variants using 2 reference panels (Methods in the online-only Data Supplement, UK Biobank genetic data).

### CMR Phenotypes

The detailed CMR protocol and analysis methods have been described previously,^9^ and further details are available in Methods in the online-only Data Supplement (UK Biobank CMR Phenotypes and Figure I in the online-only Data Supplement). Since our primary aim was to investigate the genetic basis of LV image-derived phenotypes, we excluded individuals with prevalent myocardial infarction, heart failure, or LVEF <50% to minimize the confounding influence of these preexisting conditions. Additional sample quality control measures were outlined in Methods in the online-only Data Supplement (Sample Quality Control and Figure II in the online-only Data Supplement).

### Genetic Analyses

#### Primary Analyses

The detailed analysis strategy is outlined in Methods in the online-only Data Supplement (UK Biobank Genetic Association Analysis and Figure III in the online-only Data Supplement). We estimated the heritability explained by the genotyped variants (h_g_^2^ SNP [single nucleotide polymorphism]) and bivariate genetic correlation (r_g_) using a variance component method implemented in BOLT-REML software.^10^ We next performed the discovery GWAS of each LV trait using a linear mixed-model method by BOLT-LMM software,^11^ under an additive genetic model with ~7 million imputed genetic variants with minor allele frequency ≥5% and imputation quality score >0.3. Both heritability analysis and GWAS models were adjusted for age, sex, height, weight, systolic blood pressure corrected for antihypertensive medication use (by adding 15 mm Hg), phenotype-derivation method (automatic/manual), array type (UK Biobank versus UK BiLEVE array), and imaging center (Methods in the online-only Data Supplement, Definitions of Covariates). The untransformed LV phenotypes that showed evidence of positive skewness normalized well after rank-based inverse normal transformation (Figure IV in the online-only Data Supplement). We used these rank-based inverse normal transformed phenotypes in all primary analysis models for heritability, genotypic correlation, and GWAS. In addition to consideration for the multiple testing of genotypes involved in a single GWAS, we had to consider multiple hypothesis testing with our 6 distinct, albeit correlated, LV traits. The effective number of phenotype-association tests estimated by the Galwey method was 3.3. However, we set a more stringent threshold for genome-wide significance at *P*<5×10^−8^/5=1×10^−8^ given the absence of a large replication cohort of comparable size. Genome-wide significant loci were defined by the most significant variant (known as the sentinel or lead variant) and their proxies (correlated variants) in linkage disequilibrium (LD) r^2^>0.1 in a 1 Mb region.

We also performed conditional analysis to determine the presence of secondary independent signals within the GWAS loci and nested linear regression analyses to calculate the percentage variance explained by the lead variants (Methods in the online-only Data Supplement, Conditional Analysis and Percentage Variance).

#### Secondary Analyses

As a sensitivity analysis, we adjusted the primary analysis models with additional cardiovascular risk factors, namely diastolic blood pressure corrected for antihypertensive medication use (by adding 10 mm Hg), body mass index as a measure of obesity (replacing weight in the primary model to avoid collinearity), smoking status, regular alcohol use, dyslipidemia, and diabetes mellitus (Methods in the online-only Data Supplement, Definitions of Covariates). We also repeated the association analyses with untransformed LV traits while controlling for the same covariates as the primary analysis to obtain more comprehensible effect sizes (β), which are in the same unit of measurements as the LV traits (Methods in the online-only Data Supplement, Secondary Analyses). Since the ratio of LV mass to LVEDV^0.67^ (concentricity^0.67^) was previously shown to be more correlated with both LV wall thickness and systolic blood pressure than the standard definition of concentricity (LVMVR),^12^ we repeated the association analysis with this phenotype. Furthermore, potential mediating effects of systolic blood pressure and sex were explored by incorporating the lead variant and covariate interaction terms in the primary models, using the mixed-model method implemented in the MMAP software (https://mmap.github.io/). Bonferroni correction was applied on the interaction *P* values to adjust for the number of variants tested.

#### Replication Analyses in the MESA Cohort

We performed the association analyses for all our sentinel variants in both European and non-European ancestries of the MESA study (Multi-Ethnic Study of Atherosclerosis).^13^ After sample quality control (see sample selection flowchart in Figure V in the online-only Data Supplement), a total of 4383 individuals from the MESA study (European = 1742, African American = 1083, Chinese = 586, and Hispanic = 972) were included in the look-up analysis. Further details on the design and analysis of MESA cohort are available in Methods in the online-only Data Supplement.

#### Pleiotropy Analyses

We searched PubMed to collate all genome-wide significant variants (*P*<5×10^−8^) reported in published literature on closely related phenotypes (ECHO-derived LV measurements, LV hypertrophy identified by ECG) and performed a lookup of these variants in our GWAS results. We cross-referenced our sentinel variants and their close proxies (LD r^2^≥0.8) with Phenoscanner database v2 (http://www.phenoscanner.medschl.cam.ac.uk/) and presented the variants that showed strong associations with other traits at *P*<5×10^−8^.^14^ We also interrogated the GeneAtlas database (http://geneatlas.roslin.ed.ac.uk/) to assess the associations of our variants with other traits in the UK Biobank.^15^

### Functional Annotation

We employed an integrative bioinformatics approach to compile the functional information at both variant and gene levels. Significant genomic loci were annotated using multiple lines of evidence including the presence of coding variant, gene expression data, chromatin interaction analyses, knockout models, and literature review (Methods in the online-only Data Supplement, Bioinformatic Annotation).

### Polygenic Scoring

We used the LDPred tool^16^ to construct the polygenic risk score (PRS) of each LV trait based on the effect sizes derived from the LV GWASs and predicted the risk of heart failure event in the remainder of the UK Biobank cohort. LDPred considers the tuning parameter known as the fraction of causal variant (*ρ*). To choose the best unbiased *ρ*, we first split the UK Biobank dataset into the training (2033 cases; 149 461 controls) and test (3106 cases; 224 134 controls) sets. The final PRS constructed from the best-fit *ρ* value was used to predict heart failure in the holdout test dataset using a logistic regression model controlled for age, sex, body mass index, systolic and diastolic blood pressure adjusted for antihypertensive medication use, smoking status, regular alcohol use, dyslipidemia, diabetes mellitus, and 15 genetic principal components. We also explored the prospective association between LV-PRS quintiles and incident heart failure in the test dataset using multivariate Cox proportional hazards models adjusted for the same covariates as the logistic regression models. Further information on PRS is available in Methods in the online-only Data Supplement (Polygenic Risk Prediction of Heart Failure Events).

## Results

**Figure 1.**
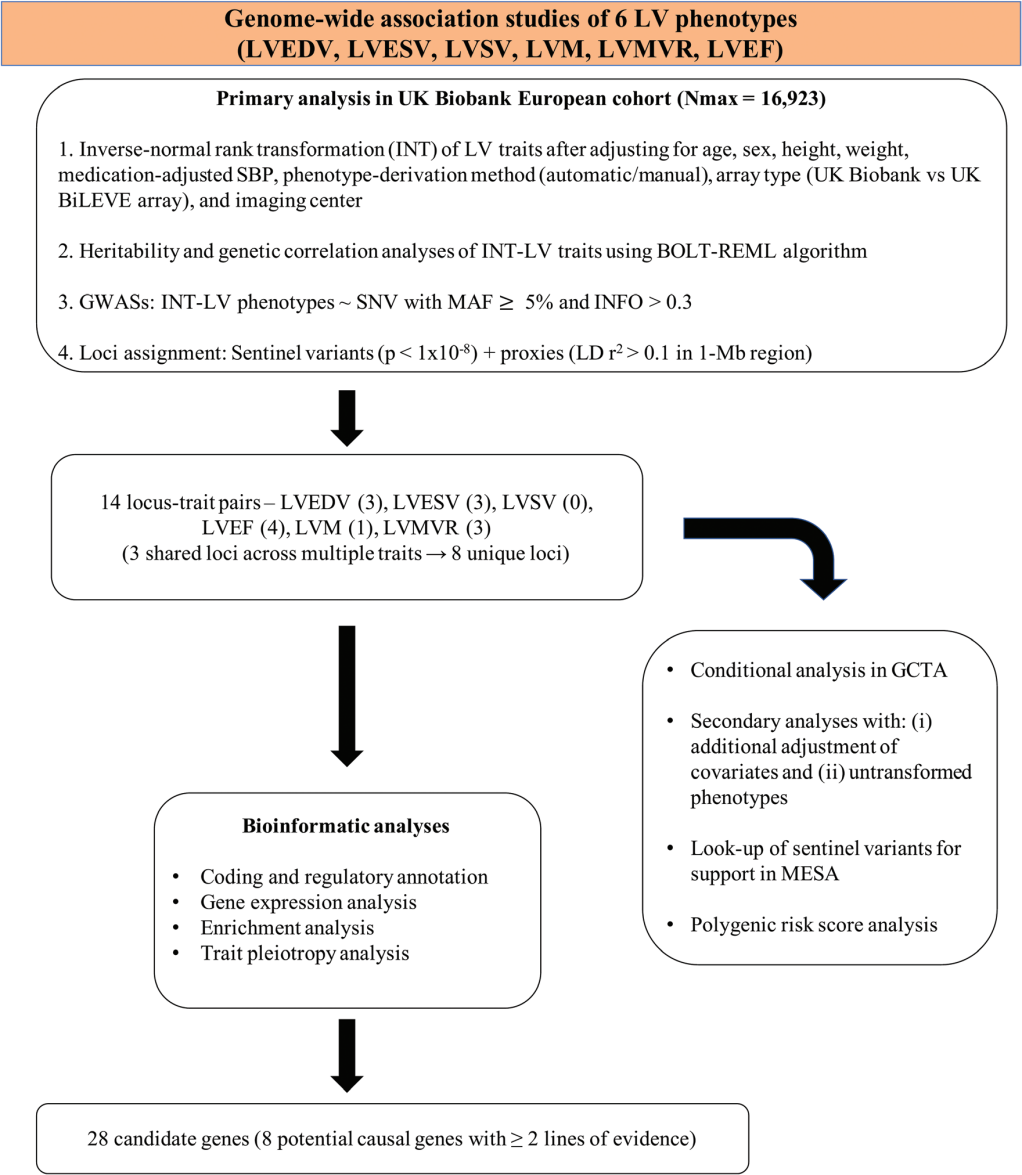
**Flowchart of analysis strategy.** GCTA indicates genome-wide complex trait analysis; GWAS, genome-wide association study; INFO, imputation quality score; INT, inverse-normal rank transformation; LD, linkage disequilibrium; LV, left ventricular; LVEDV, left ventricular end-diastolic volume; LVEF, left ventricular ejection fraction; LVESV, left ventricular end-systolic volume; LVM, left ventricular mass; LVMVR, left ventricular mass to end-diastolic volume ratio; LVSV, left ventricular stroke volume; MAF, minor allele frequency; MESA, Multi-Ethnic Study of Atherosclerosis; Nmax, maximum sample size; REML, restricted maximal likelihood; SBP, systolic blood pressure; and SNV, single nucleotide variant.

The overall study design is illustrated in Figure 1, and the summary characteristics of the UK Biobank CMR cohort are presented in Table I in the online-only Data Supplement. The mean±SD age of the cohort 62.5±7.5 years, and 45.8% were men. The primary analysis comprised a total of 16 923 European individuals with a maximum sample size of LVEDV (n=16 920), LVESV (n=16 920), LVSV (n=16 917), LVEF (n=16 923), LVM (n=16 920), and LVMVR (n=16 884). Approximately 25% of the CMR studies were manually analyzed, and the remainder were segmented by a deep learning algorithm. The reproducibility of both manual and automatic measurements was very high (intraclass correlation coefficient ranged from 0.88 to 0.98).

### Heritability and Genotypic Correlation

**Figure 2.**
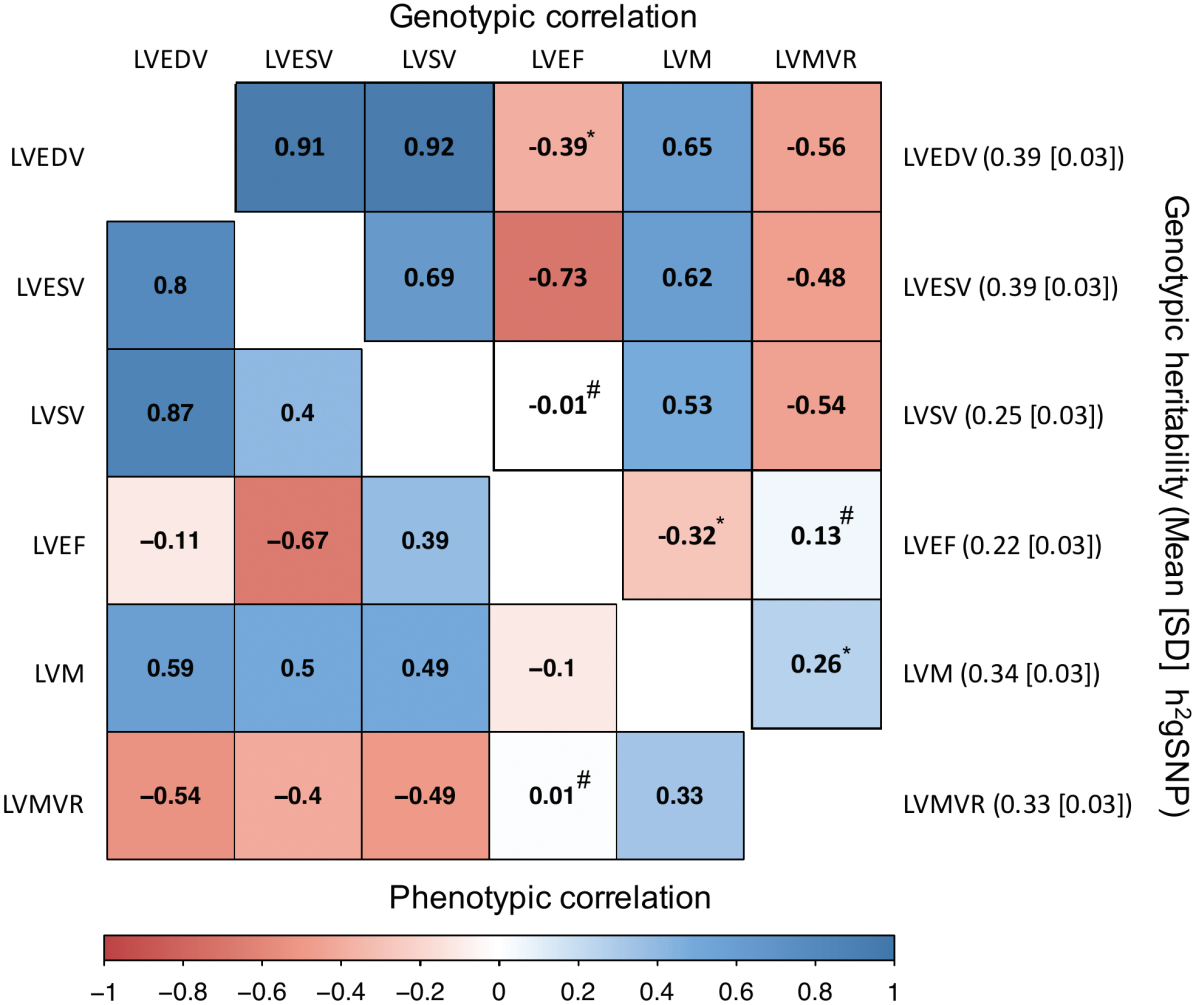
**SNP heritability and genotypic and phenotypic correlations between LV traits.** The upper triangle of correlogram represents the degree of genotypic correlation, and the lower triangle represents the degree of phenotypic correlation. Heritability estimated from genotyped SNPs is presented on the right-hand side of the figure. *P<0.0001; #*P*>0.05; All other correlation estimates had *P*<1×10^–16^. LV indicates left ventricular; LVEDV, left ventricular end-diastolic volume; LVEF, left ventricular ejection fraction; LVESV, left ventricular end-systolic volume; LVM, left ventricular mass; LVMVR, left ventricular mass to end-diastolic volume ratio; LVSV, left ventricular stroke volume; and SNP, single nucleotide polymorphism.

The highest genome-wide heritability (h^2^_g_ SNP) estimates were observed for the structural traits such as LVEDV and LVESV (both at 39%), followed by LVM at 34% and LVMVR at 33%, while the functional traits such as LVSV and LVEF had lower heritability (25% and 22%, respectively). The genotypic correlations between LV traits ranged from very high (r_g_=0.92 between LVEDV and LVSV) to very low (r_g_=−0.01 between LVSV and LVEF; Figure 2).

### Genomic Loci Associated With LV Phenotypes

**Figure 3.**
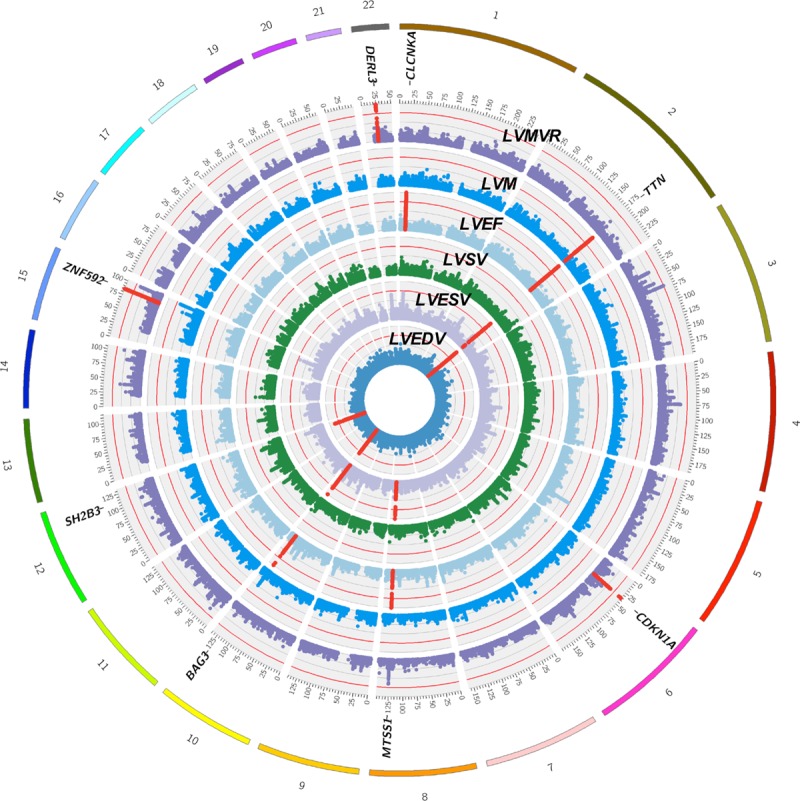
**Genomic loci associated with CMR-LV phenotypes.** Circular Manhattan plot depicting the genome-wide association study results of all LV traits. The red line indicates the genome-wide significant threshold at *P*<1×10^-8^. No genome-wide significant locus was found for LVSV. The significant genomic loci are denoted by the red dots. CMR indicates cardiovascular magnetic resonance; LV, left ventricular; LVEDV, left ventricular end-diastolic volume; LVEF, left ventricular ejection fraction; LVESV, left ventricular end-systolic volume; LVM, left ventricular mass; LVMVR, left ventricular mass to end-diastolic volume ratio; and LVSV, left ventricular stroke volume.

**Figure 4.**
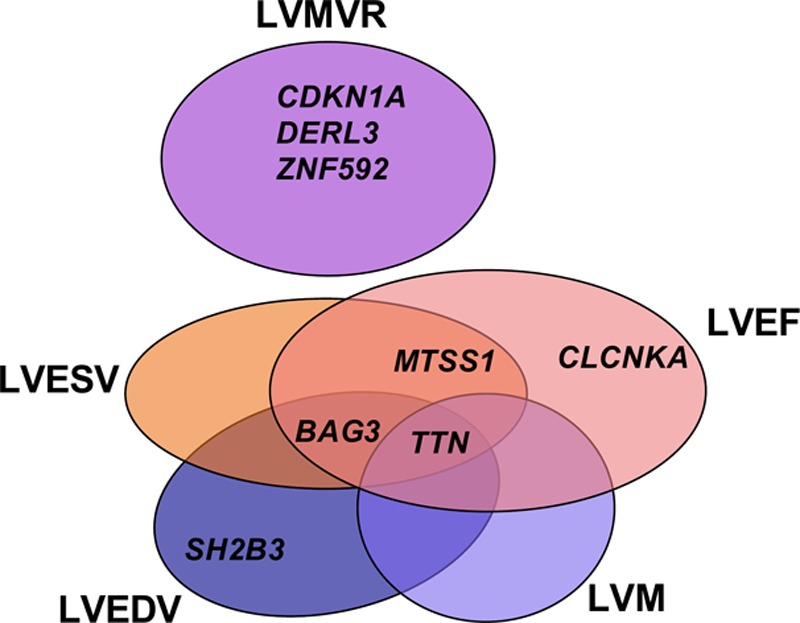
**Venn diagram of left ventricular loci.** The locus name indicates the nearest annotated gene. LVEDV indicates left ventricular end-diastolic volume; LVEF, left ventricular ejection fraction; LVESV, left ventricular end-systolic volume; LVM, left ventricular mass; and LVMVR, left ventricular mass to end-diastolic volume ratio.

We discovered a total of 14 genomic loci defined by a 1 Mb region—3 loci each for LVEDV, LVESV, and LVMVR; 4 loci for LVEF; and 1 locus for LVM—at a stringent *P*<1x10^-8^ as summarized in Table 1 and Figure 3. There was no evidence of population stratification or cryptic relatedness (genomic inflation factor, λ=1.047–1.097, quantile-quantile plots in Figure VI in the online-only Data Supplement). We assigned a single sentinel variant with the lowest GWAS *P* value for each locus and plotted the LocusZoom plots for all sentinel variants (Figure VII in the online-only Data Supplement). Variants at several loci were associated with more than 1 LV trait. The *TTN* locus was associated with 4 LV traits (LVEDV, LVESV, LVEF, LVM), and the *BAG3* and *MTSS1* loci were shared across more than 1 trait (Figure 4). The LV remodeling trait, LVMVR, had 3 distinct loci (*CDKN1A*, *DERL3*, and *ZNF592*), which were not shared with other LV traits. No significant locus was found for LVSV. In total, 8 unique loci were identified. There was no evidence of secondary independent variants achieving the prespecified criteria at any loci in conditional analyses (Table II in the online-only Data Supplement). The percentages of trait variance explained by the sentinel variants were small as expected given the limited number of significant loci for each trait (LVEDV 0.21%, LVESV 0.32%, LVEF 0.38%, LVM 0.10%, LVMVR 0.46%).

**Table 1.**
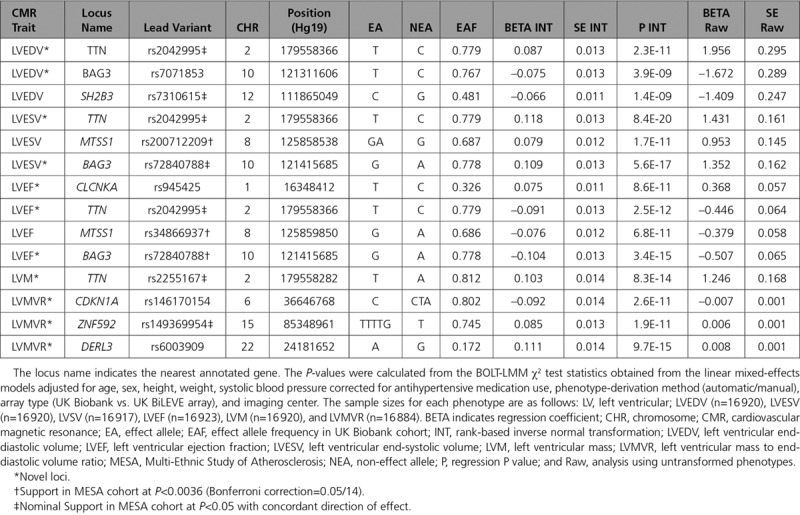
Genomic Loci Identified for CMR-Derived LV Phenotypes

### Secondary Analyses

The secondary analysis models, additionally adjusted for other cardiovascular risk factors, produced results generally congruous with the primary analyses, except for the *SH2B3* locus for LVEDV, where the GWAS *P* value of sentinel variant (rs7310615) became significantly attenuated (GWAS *P* secondary=1.5×10^−5^, −log10 *P* primary/−log10 *P* secondary=1.8; Table III in the online-only Data Supplement). The β estimates and the *P* values of our primary results and the analyses with untransformed LV phenotypes were highly correlated (ρ=0.98–1.0 for β and ρ=0.94–0.99 for *P*) with no significant attenuation of GWAS *P* values (−log10 *P* primary/−log10 *P* secondary<1.5) (Table IV in the online-only Data Supplement). The absolute effect sizes in the models with untransformed phenotypes ranged between 1 to 2 mL for LVEDV and LVESV and ~0.5% for LVEF per allele (Table 1). The lead variants for LVMVR loci remained genome-wide significant (*P*<1×10^−8^) in the sensitivity analyses with concentricity^0.67^ index. There was no significant interaction between systolic blood pressure and the lead variants (Table V in the online-only Data Supplement). In contrast, male sex significantly mediated the effects of lead variants in the *TTN* locus in the direction of larger LVEDV (interaction β=2.63 mL per allele, *P*=8.9×10^−6^) and LVESV (interaction β = 1.43 mL per allele, *P*=9.3×10^−6^) and higher LVM (interaction β = 1.42 g per allele, *P* =2.7×10^−5^) (Table VI in the online-only Data Supplement).

### Follow-Up of Loci in an Independent Multiethnic Cohort

Out of 14 locus-trait associations discovered in the UK Biobank, we validated 3 loci at Bonferroni significance (*P*<0.0036 [0.05/14]) and 7 loci at nominal significance (*P*<0.05 with concordant direction of effect) in at least 1 of the MESA ethnic subcohorts (Summary Characteristics in Table VII in the online-only Data Supplement). The rs200712209 variant associated with LVESV and rs34866937 variant associated with LVEF—both in *MTSS1* locus—were replicated in the European subset of MESA study after Bonferroni correction. Three other variants tagging *TTN*, *BAG3*, and *ZNF592* loci were nominally associated with LVESV, LVEF, and LVMVR traits in the MESA European cohort (Table VIII in the online-only Data Supplement).

### Genetic Relationships Between CMR LV Phenotypes With Other Related Traits

For ECHO traits, 2 previously reported variants in the *SH2B3* and *MTSS1* loci were genome-wide significant, and 4 other variants were nominally significant (*P*<0.05 with concordant directionality) for the corresponding CMR traits in our GWAS (Table IX in the online-only Data Supplement). The rs10774625 variant reported by Wild et al^2^ for ECHO-derived LV end-diastolic diameter was in high LD (r^2^=0.95) with the sentinel variant at *SH2B3* in our LVEDV GWAS. Previously unvalidated rs34866937 variant at the *MTSS1* locus, reported by Kanai et al for ECHO-derived LV end-systolic diameter and ECHO-derived LVEF in a Japanese population, was genome-wide significant for CMR-derived LVESV and LVEF in our European GWAS.^3^ For ECG-LV hypertrophy traits, 13 previously reported variants were nominally associated with our CMR-derived LVM (Table X in the online-only Data Supplement).

We also explored the association between our sentinel variants and their proxies with other traits from published GWASs using Phenoscanner.^14^ The variants in the *SH2B3* locus for LVEDV was associated with multiple risk factors that could mediate the cardiac remodeling process such as blood pressure/hypertension, cholesterol/low-density lipoprotein level, diabetes mellitus, and smoking status. The variants in the *CLCNKA* and *BAG3* loci for LVEF and LVESV were associated with dilated cardiomyopathy (DCM) (Table XI in the online-only Data Supplement). We also interrogated the Gene Atlas PheWAS database, which reported the association results of hundreds of traits in the UK Biobank with our sentinel variants. The sentinel variant in the *SH2B3* locus (rs7310615) was again found to be highly associated with the presence of hypertension (*P*=5.9×10^−46^) and ischemic heart disease (*P*=4.8×10^−14^) in the UK Biobank (Table XII in the online-only Data Supplement).

### Functional Annotation of Variants

At variant-level annotation, we identified a total of 238 candidate variants for all LV traits (7.6% exonic variants, 45% intronic variants, 30% intergenic variants; see Figure VIII in the online-only Data Supplement). Out of 18 exonic variants, 12 were nonsynonymous and were located in the *BAG3*, *ALPK3*, *TTN*, *SH2B3*, *NMB*, and *WDR73* genes. The missense variant, rs2234962, in the *BAG3* gene for LVESV and LVEF was predicted to be damaging by at least 2 prediction tools.

Among 220 noncoding variants, 52 were located in promotor histone marks, 148 in enhancer histone marks, and 82 in DNase I sites, and 13 altered the binding sites of regulatory proteins. We also found 6 noncoding variants that were highly conserved in vertebrates according to site-specific phylogenetic analysis (SiPhy) software.^17^ RegulomeDB database ranked 3 noncoding variants in the *ZNF592* locus as class 1f (strong support for functional importance).^18^ Of these variants, 2 (rs2175567 and rs17598603) were intronic for the *NMB* gene, and the other (rs7237) was located in the 3’ UTR region of the *WDR73* gene. The expression quantitative trait locus analysis in the GTEx (Genotype-Tissue Expression) dataset revealed that rs35006907, a close proxy of the sentinel variants in the *MTSS1* locus for LVESV and LVEF, was associated at false discovery rate <0.05 with the *MTSS1* gene expression in left ventricle and left atrial appendage tissues.^19^ Additionally, rs2070458, a proxy variant in the *DERL3* locus for LVMVR, was associated with the *MMP11* gene expression in LV tissue (Table XIII in the online-only Data Supplement).

We explored the long-range interaction influence of the sentinel variants and their proxies in the long-range chromatin interaction (Hi-C) data of aorta, left ventricle, and right ventricle, and found 11 potential target genes (Table XIV in the online-only Data Supplement). A summary of all variant-level annotations is presented in Table XV in the online-only Data Supplement. The gene prioritization, gene-set enrichment, and pathway analyses for the LV traits did not yield any significant results. However, in the tissue-specific enrichment analysis using the data from the Roadmap Epigenomics project, there was a significant enrichment of our genome-wide significant LV variants within the regions of DNase I hotspots in fetal heart tissue (Figure IX in the online-only Data Supplement).

**Figure 5.**
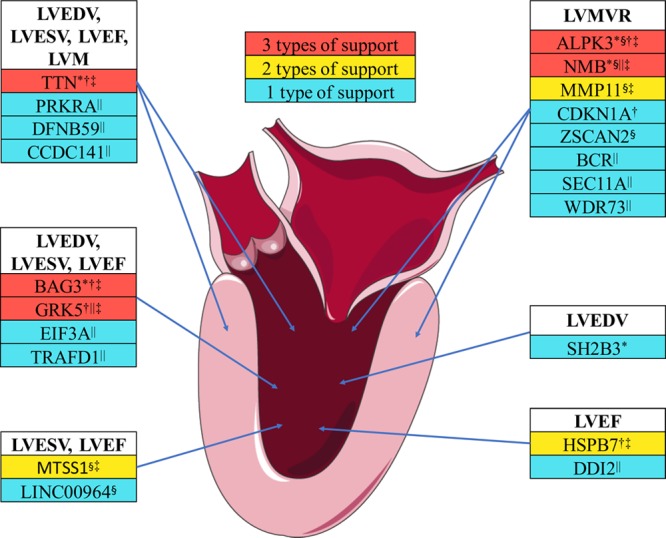
**Summary of genes associated with left ventricular traits.** Genes are ranked based on the supporting evidence summarized in Table XI in the online-only Data Supplement based on the presence of nonsynonymous variant, gene expression data, chromatin interaction analyses, literature review, and knockout models. LVEDV indicates left ventricular end-diastolic volume; LVEF, left ventricular ejection fraction; LVESV, left ventricular end-systolic volume; LVM, left ventricular mass; and LVMVR, left ventricular mass to end-diastolic volume ratio. *Coding variant gene, †knockout phenotype, ‡previously reported cardiovascular biology or strong functional rationale, §expression quantitative trait locus gene, ‖Hi-C long-range interaction gene. The illustration used elements with permission from Servier Medical Art.

At gene-level annotation, we identified a total of 28 candidate genes at 8 unique loci: 5 genes were prioritized by presence of nonsynonymous variant (sentinel or proxies with LD r^2^≥0.8), 6 were prioritized by expression quantitative trait locus data in cardiovascular tissues, 11 were prioritized based on long-range chromatin interactional analyses, and 6 were prioritized by availability of knockout models (Table XVI in the online-only Data Supplement). A summary of prioritized genes for all LV traits is depicted in Figure 5. Evaluation of these genes as input in the GeneNetwork pathway analysis revealed enrichment for terms related to heart development, regulation of the force of heart contraction, and abnormality of the cardiovascular system (Table XVII in the online-only Data Supplement).^20^

### PRS Analyses

We explored the predictive ability of PRSs derived from the genetic variants associated with LV traits to predict heart failure events in an independent test sample of the UK Biobank cohort (3106 cases, 224 134 controls). All LV-PRSs (except LVM-PRS) were significantly associated with heart failure. The PRS quintiles of LVEDV and LVESV were associated with higher odds of heart failure (odds ratio [OR] 1.25–1.41 for the top 20% versus bottom 20% group), while the opposite pattern was observed for LVEF- and LVMVR-PRSs across all levels of adjustments for potential confounders including age, sex, body size, and cardiovascular risk factors (Table 2). The sensitivity analyses including only incident cases produced similar patterns of association between LV-PRS quintiles and incidence of heart failure (Figure X in the online-only Data Supplement).

**Table 2.**
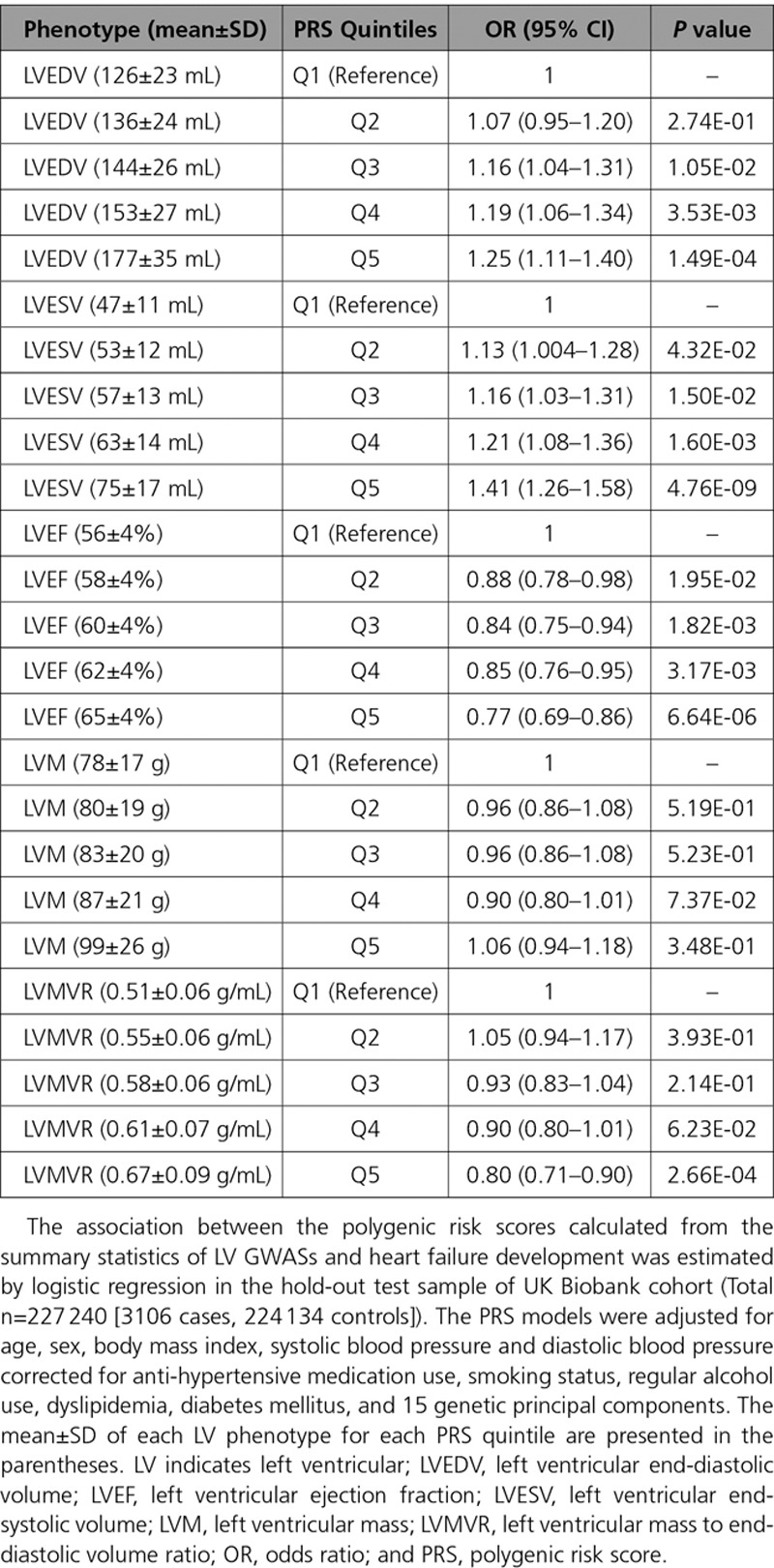
Polygenic Risk Prediction of Heart Failure Using PRSs Constructed from Variants Associated with LV Traits

## Discussion

This is the largest individual-level GWAS to date investigating the genetic architecture of prognostically important LV phenotypes derived from CMR. The key strength of this study is the combination of a standardized, highly precise, and reproducible phenotyping process with a high-quality dense genotype dataset in a cohort of ~17 000 individuals free from myocardial infarction and heart failure. This strategy yielded a total of 14 locus-trait associations. Three loci (*TTN*, *BAG3*, and *MTSS1*) were shared between more than 1 LV traits, resulting in the discovery of 8 unique loci, of which 6 were novel for LV imaging traits. The follow-up analysis of our sentinel variants in an independent multiethnic cohort (MESA) showed strong support for 2 loci (*MTSS1* and *BAG3*) at the level of Bonferroni significance, while 3 other loci (*TTN*, *SH2B3*, and *ZNF592*) achieved nominal support. Our enrichment analyses revealed that the regulatory variants associated with the LV loci are highly enriched in the fetal heart tissue, and the candidate genes for these loci are involved in the cardiac developmental pathways and regulation of the LV contractile mechanism.

Although the LV image-derived phenotypes were known to be heritable, their reported heritability varied widely, ranging from 15% to 84% for LVM, which may reflect the methods used including monozygotic and dizygotic twins, siblings, nuclear, and complex families.^21,22^ In this study, we provide a robust estimate of the proportion of LV phenotypic variance explained by the genotype (h^2^_g_ SNP heritability) in a large population of unrelated white individuals. A significant proportion of LV phenotypic variability was explained by the genotype (ranging from 22% to 39%), and the structural traits such as LVEDV or LVM had a noticeably higher heritability than more functional traits such as LVEF. The relatively lower heritability estimates in these functional traits could be due to the inflated interpersonal variations secondary to differences in the loading conditions and chronotropic and inotropic states.^23^ The heritability estimates of CMR-derived LV traits were overall higher than previously reported values in other complex cardiovascular traits such as resting heart rate (h^2^_g_=21%),^24^ and comparable ECHO traits (eg, LVM h^2^_g_=14.8% for ECHO versus 39% for CMR).^3^ However, the heritability of the ECHO trait was calculated using the summary-level data with LD score regression approach, which tends to produce lower estimates than the variance component method (which requires the individual-level data) as in our study. The pattern and magnitude of genotypic correlations between LV traits mostly mirrored the corresponding phenotypic correlations except for the relationship between LVSV and LVEF, where the genotypic correlation was absent despite a moderate phenotypic correlation (r_p_=0.39 vs r_g_=−0.01). The finding may suggest dissimilar genetic architecture between these 2 functional traits, which should be validated in an independent cohort. Most genomic loci observed in our study were specific for the LV traits except for the *SH2B3* locus (associated with LVEDV), which appeared to be highly pleiotropic and associated with multiple cardiovascular risk factors. This finding may explain the substantial weakening of the sentinel variant’s association *P* value in our secondary analysis additionally adjusted for a wider range of cardiovascular risk factors.

Among several candidate genes, we highlighted the functional roles of a few potential causal genes based on the bioinformatic analyses and literature review. The *TTN* (titin) gene emerged as a strong candidate causal gene for 4 LV traits (LVEDV, LVESV, LVEF, and LVM). The pivotal function of *TTN* in the maintenance of sarcomere assembly, stretch sensing and signaling, passive stiffness adjustment, and active force generation corresponds well with our finding of *TTN* being an important gene for both structural and functional LV imaging traits.^25^ Several titin-truncating variants have been found in 10% to 20% of DCM cases and may have a role in the pathogenesis of DCM in these individuals.^26^ These titin-truncating variants have been reported to occur at much lower frequencies in the general population (ie, 1.1% of alleles in the 1000 Genomes project),^27^ and were found to be associated with larger LV volumes.^28^ In our study including variants with minor allele frequency ≥5%, we found several missense variants (but no protein truncation variants) in the *TTN* locus (Table XIII in the online-only Data Supplement), which were all predicted to be benign by the pathogenicity prediction tools.

The *BAG3* (BCL2 associated athanogene 3) gene appears to be a likely candidate gene that is involved in modulation of the 2 volumetric traits (LVEDV and LVESV) and the derived LV functional index (LVEF). *BAG3* encodes an antiapoptotic protein expressed in the heart and skeletal muscle and serves as a cochaperone of the heat shock protein family.^29^ It is essential for the homeostasis of filamin and influences myocyte contraction through interaction with the b1-adrenegic receptor and the L-type calcium channel.^30,31^ Mutations in *BAG3* gene have been implicated in DCM pathogenesis with the myocardial tissues displaying evidence of myofibril disarray and relocation of *BAG3* protein in the sarcomeric Z-disc.^32^ In our cohort of individuals with preserved LVEF, free from known heart failure or DCM, we found a missense mutation (rs2234962, LD r^2^=0.99 with the sentinel variant) in the *BAG3* gene, which was reported to be damaging by 2 in silico prediction tools. The same missense variant has been indicated as the sentinel variant in a DCM GWAS,^33^ underlining the shared genetic basis of LV volumetric phenotypes and DCM. Our long-range chromatin interaction (Hi-C) analysis flagged another potential candidate gene at the *BAG* locus called *GRK5* (G protein-coupled receptor kinase 5). *GRK5* regulates cardiac development through the mammalian target of rapamycin (mTOR) pathway in zebrafish.^34,35^ Its expression level in the myocardium is elevated in heart failure, and a nonsynonymous polymorphism of *GRK5* appears to be protective of heart failure through inhibition of β-adrenergic receptor signaling.^36,37^ In addition, *GRK5* is a known drug target for β-blockers and antihypertensive agents (Table XIV in the online-only Data Supplement). At the *CLCNKA* locus for LVEF, we indicate *HSPB7* (heat shock protein family B member 7) as a likely candidate given its recognized role in cardiogenesis by modulating actin filament assembly and known association with heart failure development.^38–40^ Decreased cardiomyocyte proliferation and abnormal sarcomere morphology were observed in *HSPB7* knockout mice.

The *MTSS1* locus for CMR-derived LVESV and LVEF has previously been reported for comparable ECHO-derived LV traits.^2,3^ The sentinel variant at the *MTSS1* locus is in the intergenic region. However, its close proxy (rs35006907, r^2^>0.98) is located in the DNase I hypersensitive site in the cardiac tissue and is associated with expression of the *MTSS1* (metastasis suppressor protein 1) gene in LV and left atrial appendage tissues. This gene is involved in cytoskeletal signaling pathway and encodes a scaffold protein that regulates actin dynamics.^41,42^

The GWAS of remodeling phenotype, LVMVR, produced 3 genetic loci that were not shared with other LV traits. The *ALPK3* (alpha kinase 3) gene in the *ZNF592* locus is expressed in the developing heart and involves in cardiomyocyte differentiation.^43^ The *ALPK3* knockout mouse model exhibited evidence of cardiac-specific phenotypic changes such as LV dilatation and hypertrophy. Another potential candidate gene at the same locus is *NMB* (neuromedin B), which is associated with regulation of eating behavior and obesity.^44^ This gene has previously been highlighted as a candidate gene in a GWAS investigating the ECG indices of LV hypertrophy,^45^ in which the sentinel variant was moderately correlated (LD r^2^=0.39) with our LVMVR sentinel variant. Last, the proxy variant (rs2070458) of the *DERL3* locus for LVMVR was in cis-expression quantitative trait locus (cis-eQTL) with the *MMP11* (matrix metallopeptidase 11) gene expression in the left ventricle. *MMP11* belongs to the family of proteolytic enzymes that regulate extracellular matrix and play a role in the development of myocardial fibrosis and ventricular remodeling.^46,47^

Altogether, several key candidate genes in our GWAS loci (such as *TTN*, *BAG3*, and *MTSS1*) that were shared across multiple structural and functional LV traits encode essential proteins involved in the construction and maintenance of sarcomeric infrastructure. In contrast, the variation of LVMVR, the LV remodeling trait, was determined by the genetic loci containing candidate genes implicated in cardiomyocyte differentiation and extracellular matrix homeostasis.

Despite a limited number of loci found in this study, the PRSs constructed from LVEDV, LVESV, LVEF, and LVMVR were predictive of heart failure events in the remainder of the UK Biobank cohort. This finding reinforces the fundamental role of the LV imaging endo-phenotypes in the pathogenesis of heart failure. The directionality of association between PRSs and heart failure events was generally concordant with prior expectations (alleles associated with larger LVEDV and LVESV, and lower LVEF, were predictive of increased risk of heart failure) except for the PRS derived from LVMVR, where higher scores were correlated with lower odds of heart failure. LVMVR is a geometric phenotype where higher values are indicative of concentric remodeling or concentric hypertrophy of the left ventricle. Despite the conventional wisdom of higher LVMVR being associated with an increased risk of adverse cardiovascular outcomes, a prospective longitudinal study in the general population did not find a positive correlation between LVMVR and heart failure events.^7^ Furthermore, previous studies have reported that LV concentricity indicated by an increased LVMVR did not commonly lead to impaired LVEF, especially in the absence of interval myocardial infarction.^48,49^ The significant negative association between LVMVR-PRS and heart failure in this study may reflect the possible dominance of a dilatative (rather than hypertrophic) phenotype in the heart failure cases in our cohort. Interestingly, and contrary to the epidemiological evidence of association between LVM and incident heart failure,^7,50^ the LVM-PRS was not predictive of heart failure in our study. Although LVM appeared to be highly heritable in our genome-wide heritability analysis (h^2^_g_ SNP=34%), only a single locus was discovered at *P*<1×10^−8^ (percentage variance explained=0.1%). Thus, the limited statistical power may have curtailed the predictive ability of LVM-PRS.

We acknowledge some limitations in our study. Despite being the largest GWAS of CMR image-derived LV phenotypes, the relatively small discovery sample size translated to the discovery of a few loci explaining <0.5% of trait variance per trait. Additionally, the current sample size restricted our analysis to common variants with minor allele frequency ≥5%. However, the expected expansion of the CMR sample size to 100 000 in the UK Biobank, together with the highly optimized automatic image segmentation pipeline, will lead to future studies with statistical power to detect more genetic loci at a lower minor allele frequency threshold. Furthermore, the upcoming exome sequencing data from the UK Biobank may allow us to investigate the role of rarer, protein truncating, variants in a general population. Of note, 3 out of 8 unique loci discovered in the UK Biobank were not replicated in MESA. Therefore, the evidence for these susceptibility loci should be considered preliminary. Second, although we have performed a limited look-up of our loci in MESA for additional support, our findings should be formally validated in a larger cohort.

In summary, the findings from this study not only enhance our understanding of the genetic basis of prognostically important LV phenotypes in the general population but also underscore the intricate genetic relationship between these endophenotypes and the pathogenesis of heart failure syndrome, which may lead to potential novel therapeutic targets and personalized risk stratification strategy in the future.

## Acknowledgments

This research has been conducted using the UK Biobank Resource under Application 2964. The authors wish to thank all UK Biobank participants and staff.

## Sources of Funding

Dr Aung is supported by a Wellcome Trust Research Training Fellowship (203553/Z/16/Z). Drs Petersen, Neubauer, and Piechnik acknowledge the British Heart Foundation for funding the manual analysis to create a cardiovascular magnetic resonance imaging reference standard for the UK Biobank imaging resource in 5000 CMR scans (PG/14/89/31194). This work was part of the portfolio of translational research of the National Institute for Health Research Biomedical Research Centre at Barts and The London School of Medicine and Dentistry; Drs Cabrera, Barnes, Warren, Munroe, and Petersen acknowledge support from this center. Dr Petersen also acknowledges support from the “SmartHeart” Engineering and Physical Sciences Research Council program grant (EP/P001009/1). Drs Piechnik and Neubauer are supported by the Oxford National Institute for Health Research Biomedical Research Centre and the Oxford British Heart Foundation Centre of Research Excellence. This project was enabled through access to the Medical Research Council eMedLab Medical Bioinformatics infrastructure, supported by the Medical Research Council (grant No. MR/L016311/1). Dr Fung is supported by The Medical College of Saint Bartholomew’s Hospital Trust, an independent registered charity that promotes and advances medical and dental education and research at Barts and The London School of Medicine and Dentistry. The UK Biobank was established by the Wellcome Trust medical charity, the Medical Research Council, the Department of Health, the Scottish Government, and the Northwest Regional Development Agency. It has also received funding from the Welsh Assembly Government and the British Heart Foundation. MESA and the MESA SHARe project are conducted and supported by the National Heart, Lung, and Blood Institute in collaboration with MESA investigators. Support for MESA is provided by contracts HHSN268201500003I, N01-HC-95159, N01-HC-95160, N01-HC-95161, N01-HC-95162, N01-HC-95163, N01-HC-95164, N01-HC-95165, N01-HC-95166, N01-HC-95167, N01-HC-95168, N01-HC-95169, UL1-TR-000040, UL1-TR-001079, UL1-TR-001420, UL1-TR-001881, and DK063491. Funding for SHARe genotyping was provided by National Heart, Lung, and Blood Institute contract N02-HL-64278. Genotyping was performed at Affymetrix (Santa Clara, CA) and the Broad Institute of Harvard and the Massachusetts Institute of Technology (Boston, MA) using the Affymetrix Genome-Wide Human SNP Array 6.0.

## Disclosures

Dr Petersen provides consultancy to Circle Cardiovascular Imaging Inc, Calgary, Canada. The other authors report no conflicts.

## Supplementary Material
