# Supplementary material for: Genome-Wide Analysis of Left Ventricular Image-Derived Phenotypes Identifies Fourteen Loci Associated With Cardiac Morphogenesis and Heart Failure Development
Source: Circulation. 2019 Sep 25;140(16):1318–30. doi: 10.1161/CIRCULATIONAHA.119.041161 (PMC6791514; doi:10.1161/CIRCULATIONAHA.119.041161)
Supplement: Supplementary file 1 [file cir-140-1318-s001.pdf]

SUPPLEMENTAL MATERIAL

Supplemental Methods

UK Biobank genetic data

The UK Biobank Axiom^{TM} Array (825,927 markers) and UK BiLEVE Axiom^{TM} Array share 95% of contents and are designed to give genome-wide coverage of single nucleotide variants (SNVs) and insertion deletion (indels) variants. The detailed description of the array content is available online (http://www.ukbiobank.ac.uk/wp-content/uploads/2014/04/UK-Biobank-Axiom-Array-Content-Summary-2014.pdf). In addition, SNVs were imputed centrally using IMPUTE4 software (https://jmarchini.org/impute-4/) using two reference panels: (i) merged UK10K and 1000 Genome Phase 3 reference panels, and (ii) Haplotype Reference Consortium (HRC) reference panel. Imputation produced 92,693,895 autosomal SNVs, short indels and large structural variants. Genomic positions of the variants are reported in Reference Consortium Human Reference 37 (GRCh37) coordinates.

UK Biobank CMR phenotypes

All UK Biobank cardiovascular magnetic resonance (CMR) studies were acquired with a wide bore 1.5 Tesla scanner (MAGNETOM Aera, Syngo Platform VD13A, Siemens Healthcare, Erlangen, Germany). LV mass and volumes were measured from balanced steady-state free precession (bSSFP) cine short axis images (Supplemental Figure 1). Derivation of LV phenotypes followed 2 phases: (i) manual annotation of the first ~5000 CMR studies to create a reference segmented image dataset, and (ii) fully convolutional neural network (FCN) trained

with the aforementioned reference dataset to automatically analyse the remainder of CMR studies. Analysis of CMR images by FCN produced human expert-level performance in image segmentation at a fraction of time as previously described[1]. The end-diastolic phase of the cardiac cycle represents the largest LV cavity-volume while the end-systolic phase represents the smallest LV cavity-volume. LVEDV and LVESV were computed by Simpson 'disks summation' method where the sum of cross-sectional areas of the slices was multiplied by the slide thickness. LVM was calculated by multiplying the difference between total epicardial volume and endocardial volume with the myocardial density (1.05 g/m^2). LVSV, LVEF and LVMVR were defined as: LVSV = LVEDV – LVESV, LVEF = LVSV/LVEDV and LVMVR = LVM/LVEDV. Since the LV phenotypes are not normally distributed, we transformed the raw LV phenotypes by: (i) taking the residuals after regressing each LV phenotype on the covariates and (ii) applying rank-based inverse normal transformation (INT) to the residuals, prior to fitting the GWAS models.

Sample quality control

A total of 19,804 individuals with CMR imaging data were initially considered (manual analysis N = 5,065; automated analysis N = 14,739). 395 individuals were removed due to poor CMR image quality caused by incomplete coverage of left ventricle or image artefacts. For automatically analysed CMR studies, the segmentation quality of images with outlying measurements, defined as values more than three interquartile range (IQR) above the first quartile or below the third quartile, was visually reviewed by a European Association of Cardiovascular Imaging (EACVI) CMR level 3 certified analyst (N.A.).

We identified ancestry clusters by applying k-means clustering algorithm in R[2] (version 3.4.3) with k=4 on genotypic principal component 1 and 2 (PC 1 and PC 2) separately. The number of clusters (k) was chosen as 4 to represent the 4 main ethnic groups within the UK Biobank: White, African, Asian and Chinese. An overall clustering was carried out by intersection of PC1-4means-clusters and PC2-4means-clusters. The largest overlapping cluster represents the European (White) ancestry while discordant clustering between PC1 and PC2 represents 'Mixed/Other' category. European ancestry was ascertained only if the self-reported ethnicity agreed with k-means clustering results. In addition to restricting the sample to European ancestry, we excluded individuals with self-reported or hospital diagnosed heart failure or myocardial infarction, extreme phenotypic outliers, LVEF < 50% and missing covariates. The overview of sample selection process is presented in Supplemental Figure 2.

UK Biobank genetic association analysis

For the heritability and genetic correlation analyses, a high-quality set of model SNVs were selected using the following criteria: minor allele frequency (MAF) > 1%, a Hardy–Weinberg equilibrium (HWE) p-value = 1×10^{-6}, and missingness < 0.0015. We excluded the human leukocyte antigen (HLA) region (Chr 6: 28Mb – 34Mb) from our analyses due to its complex linkage disequilibrium (LD) structure. The same set of model SNVs as heritability analysis was used to account for the random effects in the linear mixed-models for the genetic association analyses.

Definitions of covariates

All covariates recorded at the imaging visit were used in the analysis where possible. Standing height (UK Biobank Data-Field 50) was measured with a Seca 202 device. Any missing value in height and weight data was substituted by the estimated height and weight values prior to imaging stages (UK Biobank Data-Fields 12144 and 12143, respectively). Body mass index (BMI) was calculated as: weight(kg) / Height(m)^{2}. Systolic and diastolic blood pressure (SBP and DBP) were measured several times during the imaging visit using Omron 705 IT electronic blood pressure monitor and manual sphygmomanometer. We averaged the automated and manual blood pressure readings (UK Biobank Data-Fields 4079, 4080, 93 and 94). The missing blood pressure values were replaced by the averaged systolic and diastolic brachial blood pressure measurements taken during pulse wave analysis at the imaging visit (UK Biobank Data-Fields 12674, 12675, 12697 and 12698). Participants taking blood pressure medications were identified from the union of those reported to be taking 'Blood pressure medication' in the questionnaire (UK Biobank Data-Fields 6153 and 6177) and those taking medications with known blood pressure lowering effect from the verbal interview (UK Biobank Data-Field 20004). SBP and DBP were adjusted for anti-hypertensive medication use by addition of 15mmHg and 10mmHg, respectively. Smoking status was categorised into 'Current', 'Previous' or 'Never' smokers obtained from UK Biobank Data-Field 20116. Regular alcohol use (Yes/No) was defined as consumption of alcohol at least three times per week (UK Biobank Data-Field 1558). Presence of dyslipidaemia and diabetes were ascertained from standardised questionnaires and verbal interview (UK Biobank Data-Fields 20002 and 2443) together with the data on the use of lipid-lowering medication and insulin (UK Biobank Data-Fields 6153 and 6177).

Prevalent heart failure and myocardial infarction (MI) were identified from self-reported questionnaires (UK Biobank Data-Field 20002) and main and secondary ICD10 codes (UK

Biobank Data-Fields 41202 and 41204, respectively). For MI diagnoses, the algorithmically-defined myocardial infarction outcomes (UK Biobank Data-Fields 42000 to 42005) were additionally used. Prevalence was ascertained by cross-checking the hospital admission dates against the date of visit to the imaging centre.

Conditional analysis

To determine the presence of independent secondary signals within the genome-wide significant loci, we used a conditional analysis (using --cojo-cond command) implemented in genome-wide complex trait analysis (GCTA) tool[3]. A secondary signal would be declared if: (i) the original p value of newly identified variant was lower than 1×10^{-6}; (ii) there was less than a 1.5-fold difference between the lead SNV and secondary association p values on a –log10 scale, i.e., if –log10(p lead)/-log10(p sec) < 1.5; and (iii) if there was less than a 1.5-fold difference between the main association and conditional association p values on a –log10 scale, i.e., if –log10(p sec)/-log10(p cond) < 1.5.

Percentage variance

We built two linear regression models by: (i) regressing each LV trait on all covariates in the primary model and the genome-wide significant variants for the trait and (ii) regressing each LV trait on all covariates in the primary model only. The proportion of variance explained by the genome-wide significant variants was given by the difference between the adjusted R^2 of these two models.

Secondary analyses

We evaluated the findings from the secondary analyses by: (i) calculating Spearman's rank correlation coefficients between the β estimates and the p values of the primary and secondary GWAS results and (ii) assessing if there was less than a 1.5-fold difference between the main association and secondary association p values on a -log10 scale, i.e., if –log10(p prim)/-log10(p sec) < 1.5.

Multi-Ethnic Study of Atherosclerosis (MESA)

(i) Study Design

MESA is a longitudinal study of subclinical cardiovascular disease and risk factors that predict progression to clinically overt cardiovascular disease or progression of the subclinical disease[4]. Between 2000 and 2002, MESA recruited 6,814 men and women 45 to 84 years of age from Forsyth County, North Carolina; New York City, New York; Baltimore, Maryland; St. Paul, Minnesota; Chicago, Illinois; and Los Angeles, California. Exclusion criteria were clinical cardiovascular disease, weight exceeding 136 kg (300 lb.), pregnancy, and impediment to long-term participation.

(ii) Left Ventricular traits in MESA

The following left ventricular traits were derived from fast gradient recovery echo (fGRE) CMR pulse sequence:

1. LV mass (g)
2. LV end-diastolic volume (ml)

3. LV end-systolic volume (ml)
4. LV stroke volume (ml)
5. LV ejection fraction (%)
6. LV mass to end-diastolic volume ratio (g/ml)

(iii) Additional phenotyping of MESA participants

Height was measured to the nearest 0.1 cm with the subject in stocking feet and weight was measured to the nearest pound with the subject in light clothing using a balanced scale. Systolic blood pressure was measured from oscillometric cuff and adjusted for anti-hypertensive use by adding 15 mmHg[5].

(iv) Genotype Data

Participants in the original MESA cohort, the MESA Family Study and the MESA Air Pollution Study who consented to genetic analyses were genotyped in 2009 using the Affymetrix Human SNP array 6.0. Genotype quality control (QC) for these data included filter on SNP level call rate < 95%, individual level call rate < 95%, heterozygosity > 53% as described previously. The cleaned genotypic data was deposited with MESA phenotypic data into dbGaP as the MESA SHARe project (study accession phs000209); 8,224 consenting individuals (2,685 White, 2,588 non-Hispanic African-American, 2,174 Hispanic, 777 Chinese) were included, with 897,981 variants passing study specific QC (MESA SHARe included an ancillary study, MESA Family, with approximately 1600 additional African-American and Hispanic individuals). For GWAS, the University of Michigan imputation

server was used for pre-phasing and imputation of the MESA SHARe participants using the 1,000 Genomes Phase 3 integrated variant set.

(v) Principal component analysis

We performed principal component analysis (PCA) to adjust for population structure among MESA participants, as described previously[6]. We constructed subsets of typed SNVs, thinned for LD within each race/ethnic group. We performed PCA as implemented in the program SMARTPCA[7,8] for unrelated subsets of individuals, removing inferred first-degree relatives from the analysis. We constructed histograms and QQ-plots to assess symmetry and normality of the distribution of loadings for each of the resulting principal components (PCs) to determine the optimal number of PCs for genetic association analysis.

(vi) Genetic association analysis

Among the MESA participants with LV phenotypes available, we stratified by race/ethnic group and constructed a subset of unrelated individuals by retaining at most one individual from each family. We further excluded those individuals with top PCs of ancestry > 3.5 SD from the mean within any race/ethnic group. Based on our examination of PCs within each race/ethnic group, we used 3 PCs for analysis of Whites, 1 PC for African Americans, 3 PCs for Hispanics, and 1 PC for Chinese. The sample selection procedure in the MESA cohort is outlined in Supplemental Figure 5. The analyses of LV traits were stratified by ethnic groups on the rank-based inverse normal transformed residuals model. We included covariate adjustment for age, sex, height, weight, medication-adjusted SBP, study site and PCs.

ProbABELv0.5.0[9] was used to conduct genetic association analysis using linear models with robust standard errors.

Bioinformatic annotation

We used ANNOVAR[10] to characterise all sentinel variants and their proxies in LD $r^2 \geq 0.8$ for variant location, distance to the nearest genes, amino acid substitution and functional impact based on a range of prediction tools including SIFT and PolyPhen-2. The nonsynonymous variants were classified as 'damaging' if two or more methods predicted detrimental effects and 'probably damaging' if indicated by a single prediction tool. We used HaploReg[11] to annotate the non-coding variants using supporting information from chromatin state, conservation and protein-binding data. RegulomeDB[12] was used to further prioritise the regulatory variants.

We obtained the expression quantitative trait loci (eQTL) association data of all variants in LD $r^2 \geq 0.8$ with our sentinel variants from GTEx[13] dataset v7 (https://gtexportal.org/home/). We reported the variants significantly associated with gene expression (false discovery rate, FDR < 0.05) in all tissues and further highlighted the eQTL associations in cardiovascular tissues (heart, aorta, coronary and tibial arteries). We identified potential target genes by performing the long-range chromatin interaction (Hi-C) analysis in FUMA[14] (Functional Mapping and Annotation) web-based platform v1.3.3c. The Hi-C data from left and right ventricles and aorta were obtained from the pre-processed chromatin loops computed by Fit-Hi-C pipeline[15]. We only considered the genes which showed significant promotor interactions (at FDR $< 1 \times 10^{-6}$) with the regulatory variants (RegulomeDB score ≤ 5) in LD $r^2 \geq 0.8$ with our sentinel variants. For gene prioritisation and gene-set enrichment analyses, we

entered all variants with suggestive significance (GWAS p values < $1x10^{-5}$) into DEPICT (Data-driven Expression-Prioritised Integration for Complex Traits) tool[16]. We also investigated these variants for significant enrichment within DNase I sensitive sites identified from ENCODE and Roadmap Epigenomics projects using FORGE[17] (Functional element Overlap analysis of the Results of Genome Wide Association Study) v1.1.

At gene-level annotation, we mapped all genes which are located within 10kb from our sentinel variants using BEDtools[18]. We ranked the candidate genes using multiple lines of evidence including cardiovascular tissue-specific gene expression and Hi-C data, availability of knockout phenotype in animal models (from International Mouse Phenotyping Consortium [http://www.mousephenotype.org/] and the Mouse Genome Informatics database [http://www.informatics.jax.org/]). The druggability of potential causal genes were assessed by accessing the drug-gene interaction database (http://www.dgidb.org/). We evaluated our candidate genes with GeneNetwork[19] (https://www.genenetwork.nl/) which uses gene co-regulation data, to predict pathway membership and HPO (human phenotype ontology) term associations. We next carried out an extensive literature review of all candidate genes by accessing information from GeneCards[20] (https://www.genecards.org/), OMIM (Online Mendelian Inheritance in Man) (https://www.omim.org/) and PubMed.

Polygenic risk prediction of heart failure events

Heart failure cases were identified from self-reported questionnaires (UK Biobank Data-Field 20002) and main and secondary ICD10 codes – I50.0 (Congestive heart failure), I50.1 (Left ventricular failure, unspecified), I50.9 (Heart failure, unspecified), I11.0 (Hypertensive heart disease with (congestive) heart failure), I13.0 (Hypertensive heart and renal disease with

(congestive) heart failure), I13.2 (Hypertensive heart and renal disease with both (congestive) heart failure and renal failure), I42.0 (Dilated cardiomyopathy) – in UK Biobank Data-Fields 41202 and 41204, respectively. Since the germline DNA variation had been determined prior to disease onset, we included both incident and prevalent cases in the genetic risk score (PRS) analysis. In sensitivity analysis, we excluded individuals with prevalent heart failure and calculated the hazard ratios of incident heart failure for PRS quintiles. PRS was calculated by the summation of the alleles associated with each LV trait weighted by their effect sizes ($\text{PRS} = \sum_i S_i \times G_i$, where S_i is the effect size of the effect allele and G_i is the number of the observed effective allele). We removed individuals included in the LV GWASs and their relatives (third-degree or closer; Kinship coefficient ≥ 0.044) from our target sample to get unbiased estimates of association between PRS and heart failure outcome. The analysis was restricted to ~1.2 million HapMap3 variants as recommended by the LDPred[21] authors. LDPred deploys a Bayesian method to infer the posterior mean effect size of each variant based on a genetic architecture prior and LD information. The priors drawn from the point-normal mixture distribution consider the tuning parameter known as the fraction of causal variant ($\boldsymbol{\rho}$) which were selected from a range of values using the training dataset. A range of $\boldsymbol{\rho}$ values (1.0, 0.3, 0.1, 0.03, 0.01, 0.003, and 0.001) were considered. The best $\boldsymbol{\rho}$ value was selected based on the largest Nagelkerke R^2 value in the logistic regression models of PRS predicting heart failure in the training set.

Supplemental Figures

Supplementary Figure 1. Exemplary segmented left ventricular short axis images

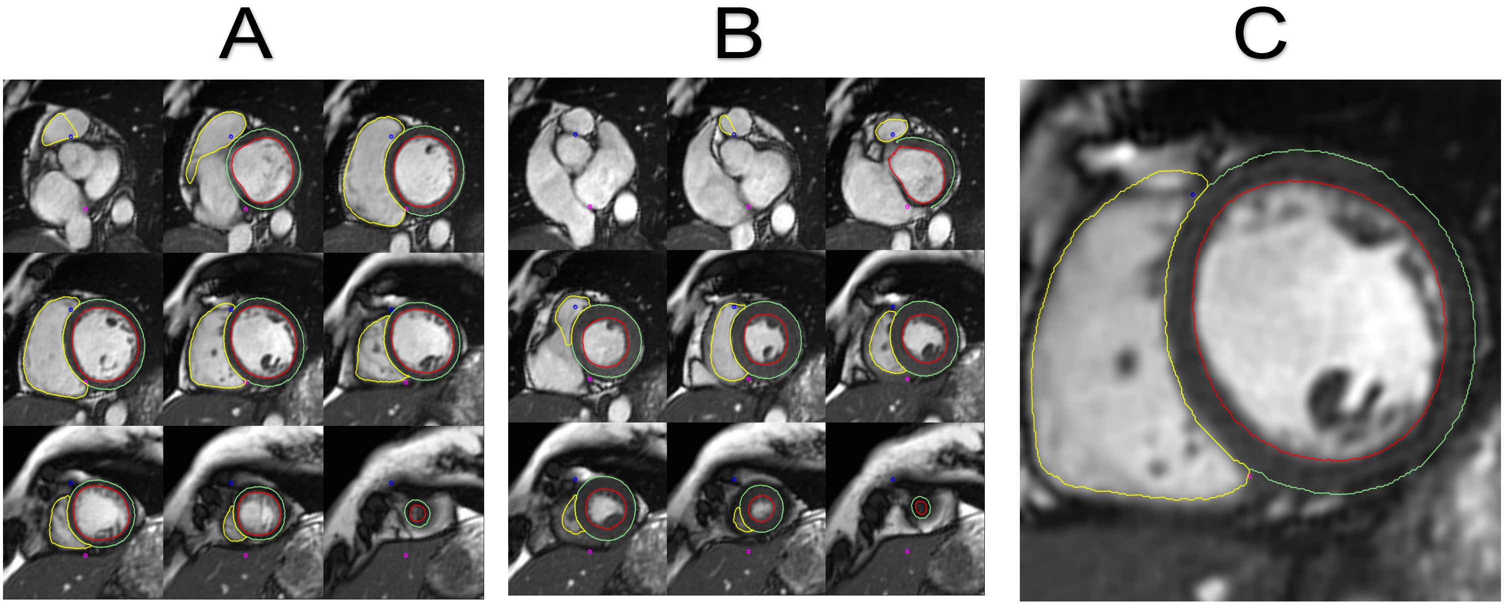

Panels A and B demonstrate the contouring of left and right ventricle from base to apex at end-diastole and end-systole, respectively. Panel C shows the zoomed-in mid-ventricular slice of left and right ventricles. The red and green contours demarcate the left ventricular endocardial and epicardial borders, respectively, while the yellow contour demarcates the right ventricular endocardial border.

Supplemental Figure 2. Sample selection flowchart

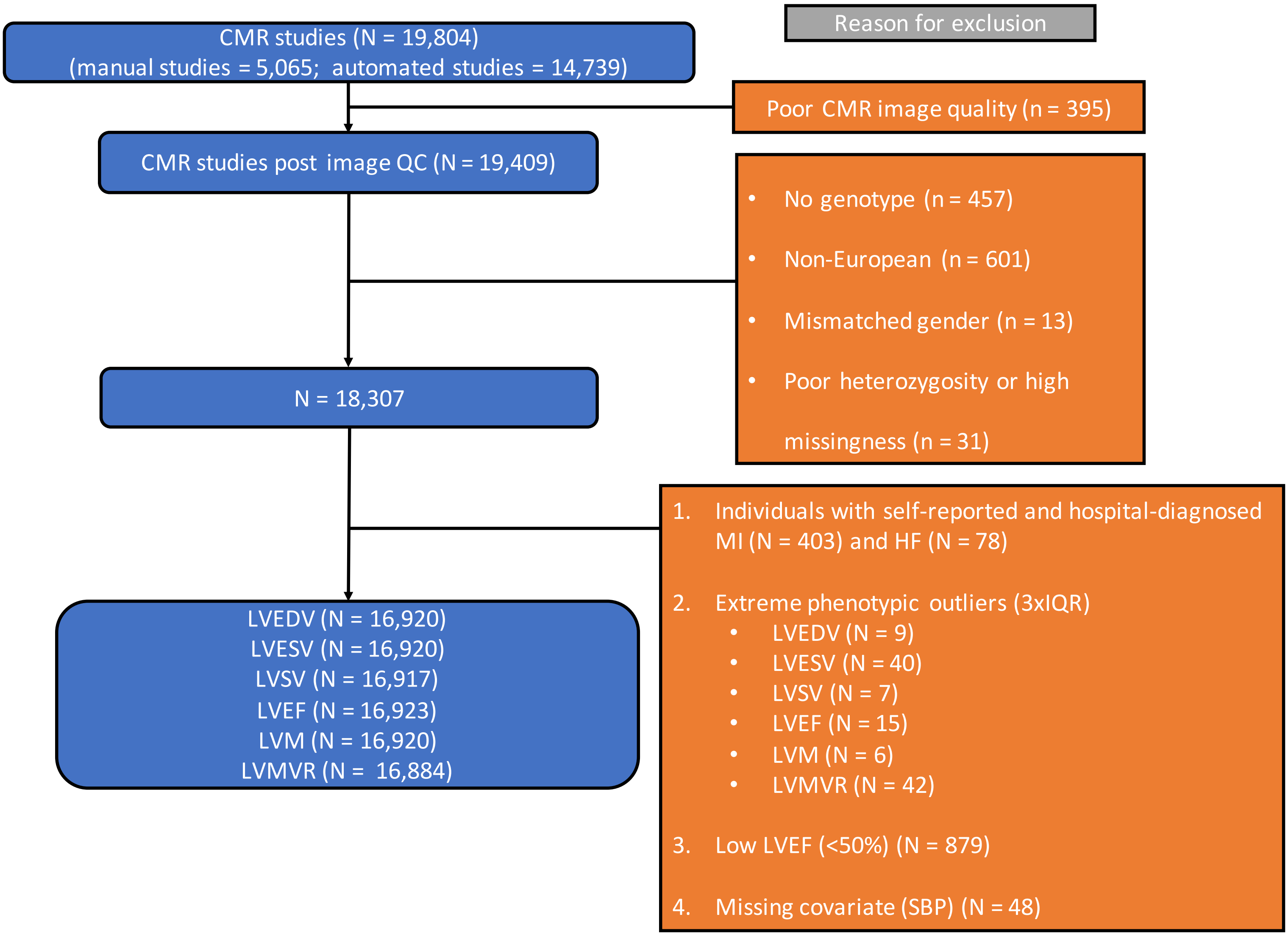

CMR, cardiovascular magnetic resonance; LVEDV, left ventricular end-diastolic volume; LVESV, left ventricular end-systolic volume; LVSV, left ventricular stroke volume; LVEF, left ventricular ejection fraction; LVM, left ventricular mass; LVMVR, left ventricular mass to end-diastolic volume ratio; MI, myocardial infarction; HF, heart failure

Supplemental Figure 3. Detailed flowchart of analysis strategy

Genome-wide association studies of 6 LV phenotypes
(LVEDV, LVESV, LVSV, LVM, LVMVR, LVEF)

Primary analysis in UK Biobank European cohort (Nmax = 16,923)

1. Inverse-normal rank transformation (IVNT) of LV traits

(i) Obtain the residuals after regressing each LV phenotype on the covariates (age, sex, height, weight, SBP adjusted for anti-hypertensive medication use, phenotype-derivation method (automatic/manual), array type (UK Biobank vs UK BiLEVE array), and imaging centre)
(ii) Apply IVNT to the residuals

2. Heritability and genetic correlation analyses of IVNT-LV traits using BOLT-REML algorithm

3. GWASs: IVNT-LV phenotypes ~ SNV with MAF ≥ 5% and INFO > 0.3

4. Loci assignment: Sentinel genome-wide significant variant ($p < 1x10^{-8}$) + proxies (LD $r^2 >$ 0.1 in 1-Mb region)

14 locus-trait pairs (3 loci shared loci across multiple traits → 8 unique loci)

Conditional analysis

Evaluation secondary independent signal in 1-Mb region from sentinel variants using GCTA "cojo-cond" command

Secondary analyses

1. GWASs: Untransformed LV phenotype ~ SNV + covariates in the primary model

2. Primary GWAS models + additional adjustment for DBP-adjusted for anti-hypertensive use, BMI, smoking status, regular alcohol use, dyslipidaemia and diabetes

Lookup in MESA study

Sentinel GWS variants (N = 11) were looked up for support in each ancestry group of MESA study (European=1,742; African-American=1,083; Hispanic=972; Chinese=586)

Polygenic risk score analyses

Polygenic prediction of heart failure outcome in the remaining (non-CMR) UK Biobank cohort (cases / controls N = 5137 / 358,879) by genetic risk scores constructed from the LV GWAS summary statistics

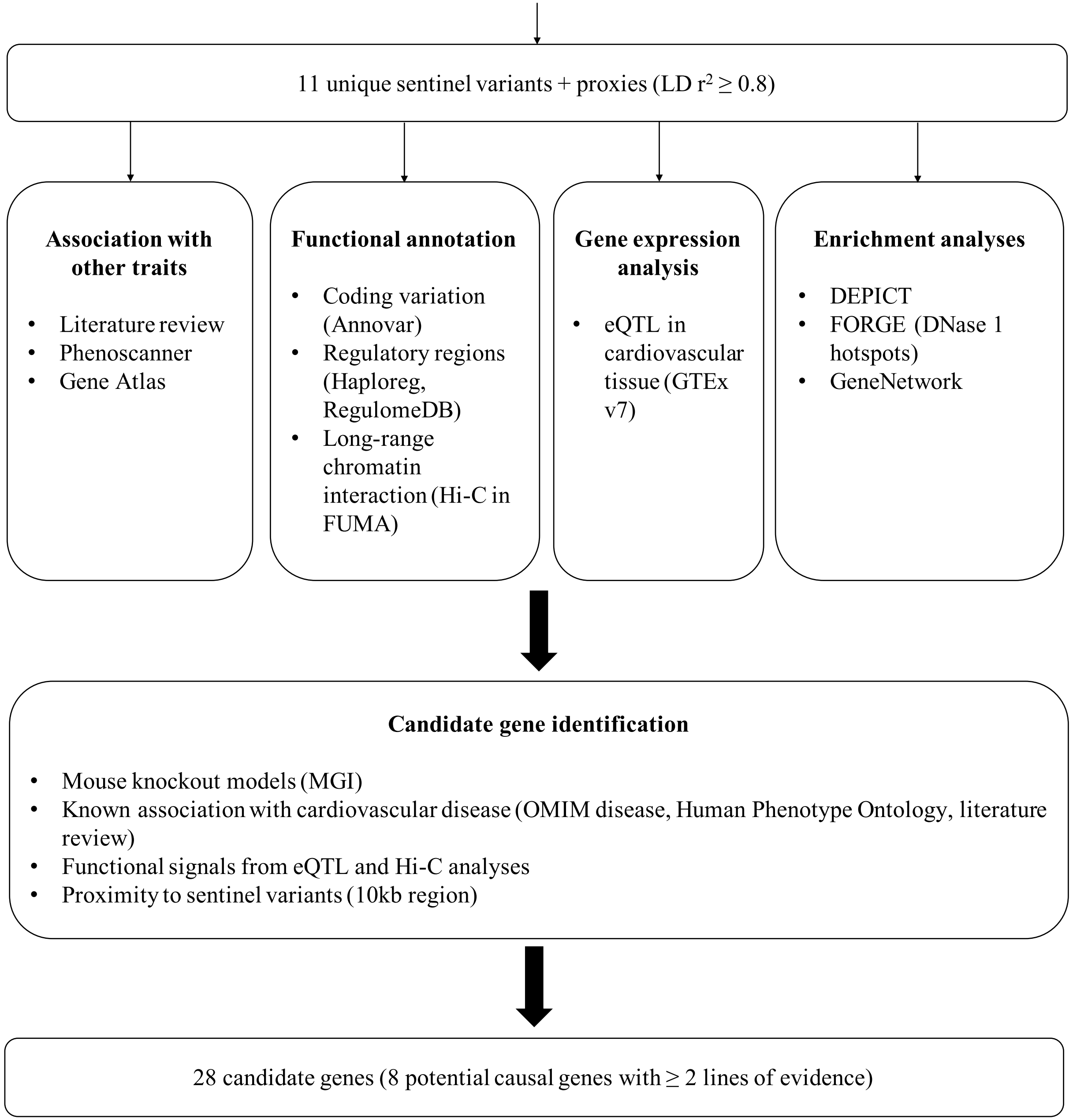
11 unique sentinel variants + proxies (LD $r^2 \geq 0.8$)
Association with other traits
• Literature review
• Phenoscanner
• Gene Atlas
Functional annotation
• Coding variation (Annovar)
• Regulatory regions (Haploreg, RegulomeDB)
• Long-range chromatin interaction (Hi-C in FUMA)
Gene expression analysis
• eQTL in cardiovascular tissue (GTEx v7)
Enrichment analyses
• DEPICT
• FORGE (DNase 1 hotspots)
• GeneNetwork
Candidate gene identification
• Mouse knockout models (MGI)
• Known association with cardiovascular disease (OMIM disease, Human Phenotype Ontology, literature review)
• Functional signals from eQTL and Hi-C analyses
• Proximity to sentinel variants (10kb region)
28 candidate genes (8 potential causal genes with ≥ 2 lines of evidence)

LVEDV, left ventricular end-diastolic volume; LVESV, left ventricular end-systolic volume; LVSV, left ventricular stroke volume; LVM, left ventricular mass; LVMVR, left ventricular mass to end-diastolic volume ratio; LVEF, left ventricular ejection fraction; SNV, single nucleotide variant; REML, restricted maximal likelihood; SBP, systolic blood pressure; MAF, minor allelic frequency; INFO, imputation quality score; LD, linkage disequilibrium; GCTA, genome-wide complex trait analysis; DBP, diastolic blood pressure; BMI, body mass index; CMR, cardiovascular magnetic resonance imaging; MESA, multi-ethnic study of atherosclerosis; Hi-C, long-range chromatin interaction analysis; FUMA, Functional Mapping and Annotation tool; eQTL, expression quantitative trait loci; GTEx, Genotype-Tissue Expression database; DEPICT, Data-driven Expression-Prioritised Integration for Complex Traits; FORGE, Functional element Overlap analysis of the Results of Genome Wide Association Study; MGI, Mouse Genome Informatics database; OMIM, Online Mendelian Inheritance in Man

Supplemental Figure 4. Histogram of left ventricular CMR phenotypes with superimposed density line

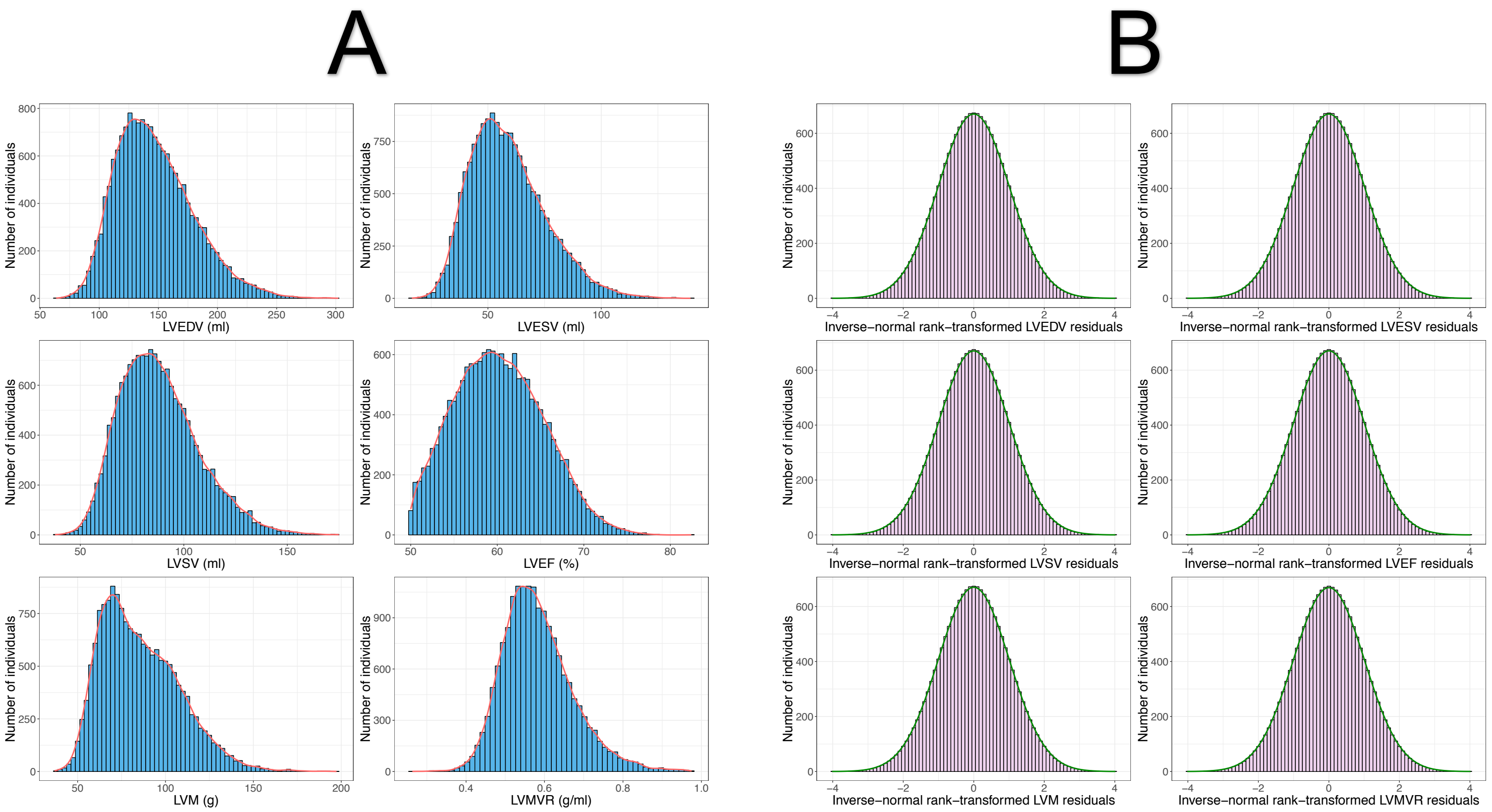

Panel A shows the distribution of untransformed LV phenotypes and Panel B shows the distribution of inverse-normal rank-transformed residuals of phenotypes after regressing on covariates.

CMR, cardiovascular magnetic resonance imaging; LVEDV, left ventricular end-diastolic volume; LVESV, left ventricular end-systolic volume; LVSV, left ventricular stroke volume; LVEF, left ventricular ejection fraction; LVM, left ventricular mass; LVMVR, left ventricular mass to end-diastolic volume ratio

Supplemental Figure 5. Sample selection flowchart in the MESA cohort

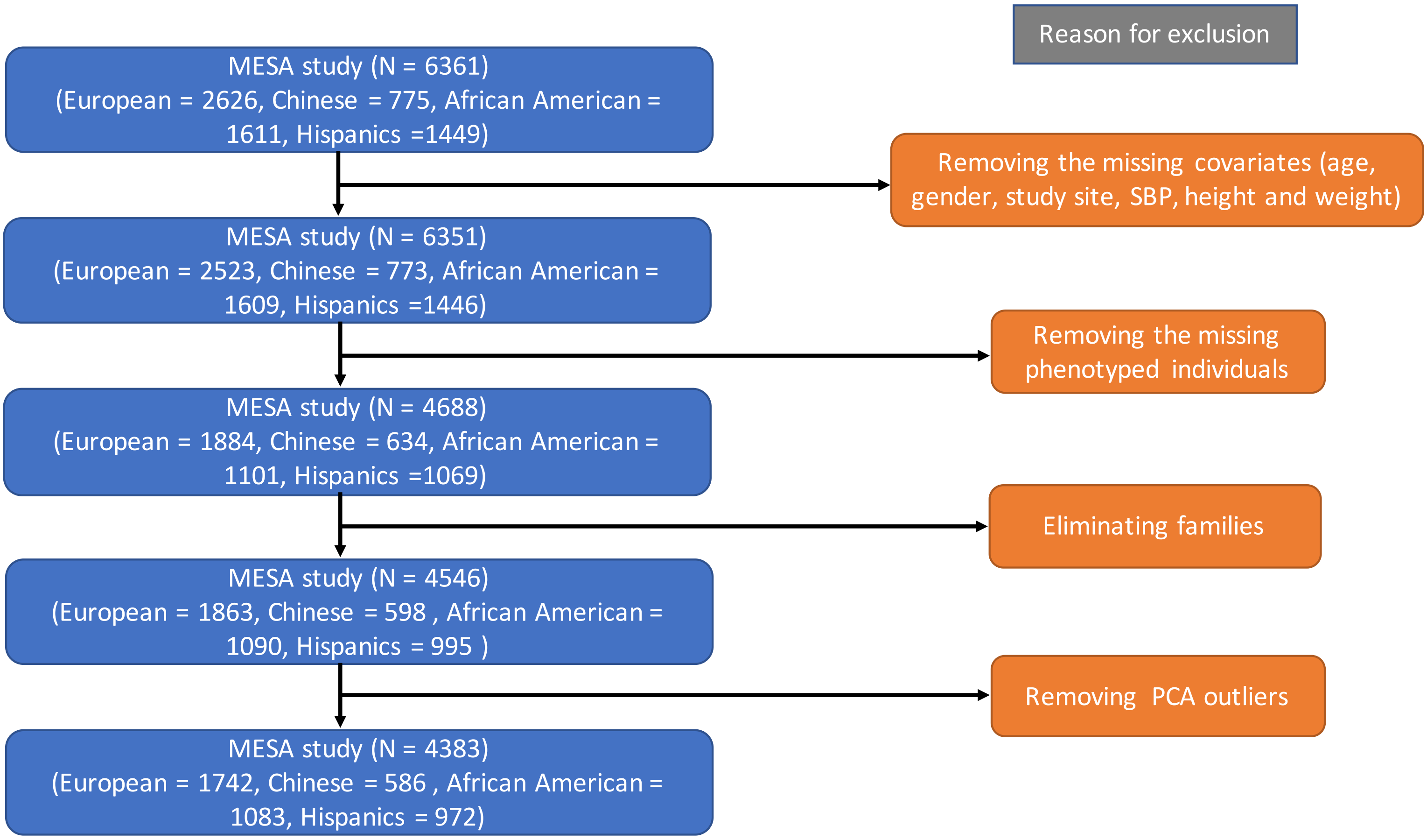

MESA, Multi-Ethnic Study of Atherosclerosis; SBP, systolic blood pressure; PCA, principal component analysis

Supplemental Figure 6. Quantile-quantile plots of GWAS p values

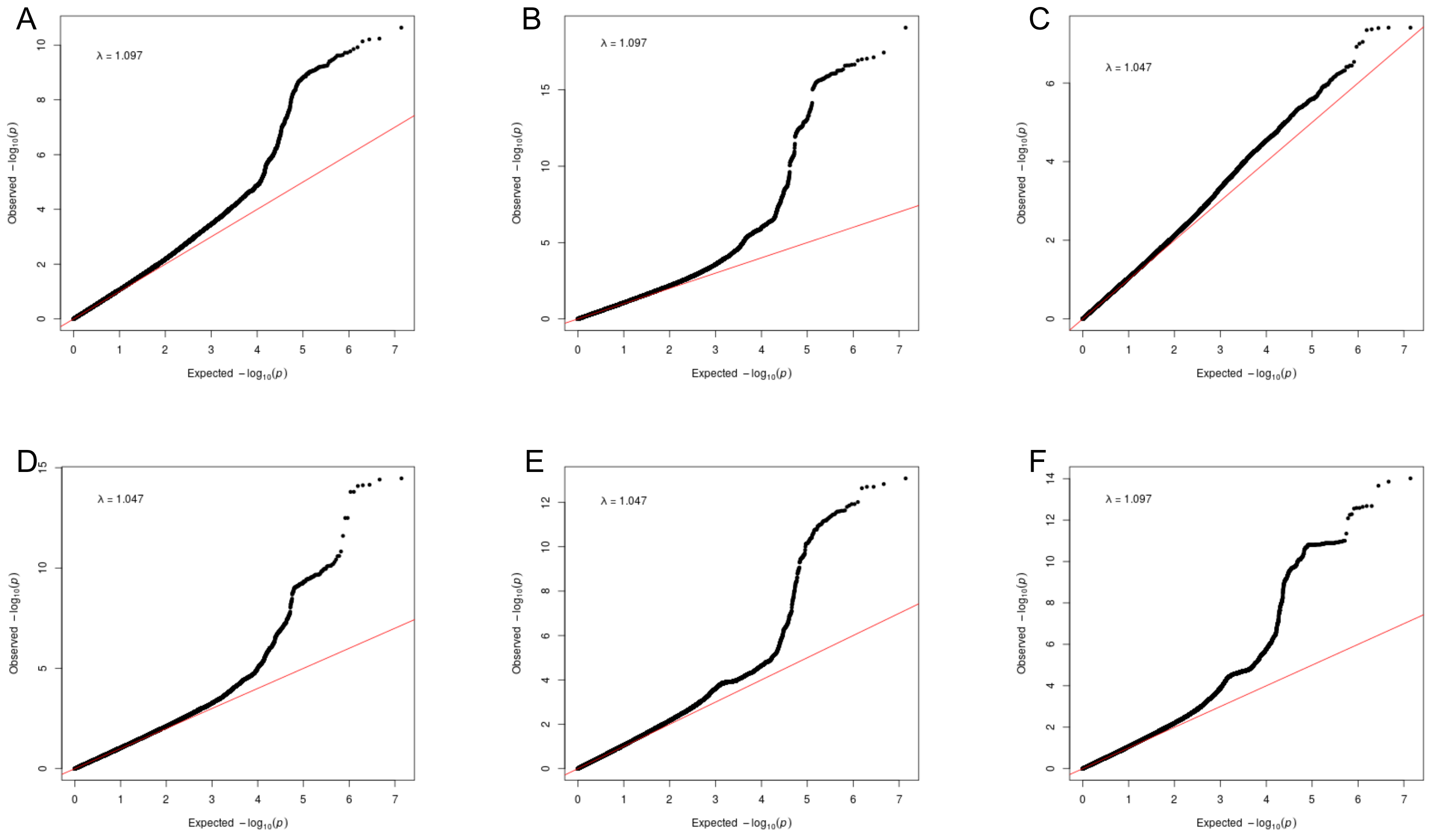

Panel A. left ventricular end-diastolic volume (LVEDV); Panel B. left ventricular end-systolic volume (LVESV); Panel C. left ventricular stroke volume (LVSV); Panel D. left ventricular ejection fraction (LVEF); Panel E. left ventricular mass (LVM); Panel F. left ventricular mass to end-diastolic volume ratio (LVMVR)

λ, genomic inflation factor

Supplemental Figure 7. LocusZoom plots of LV traits

LVEDV

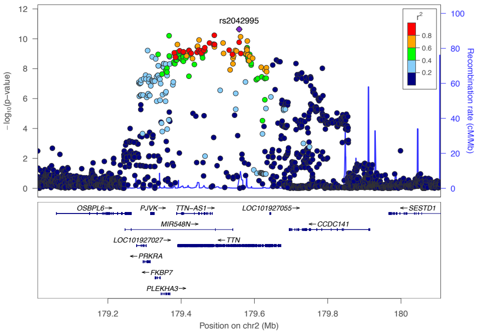

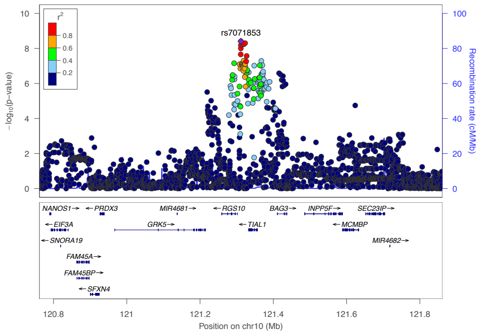

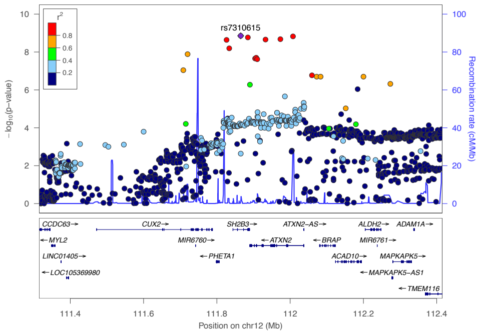

LVESV

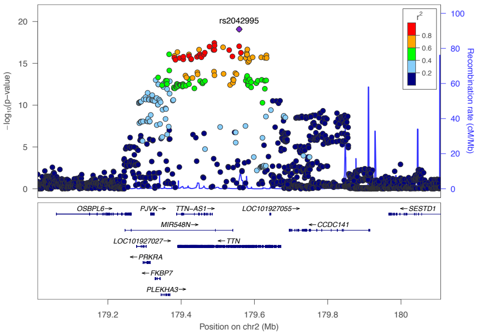

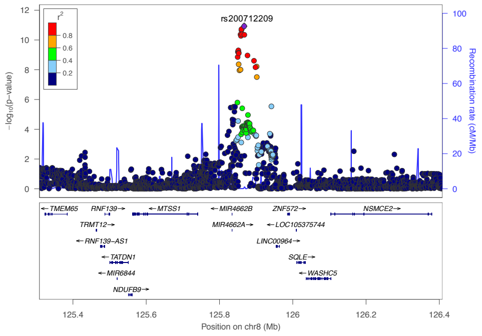

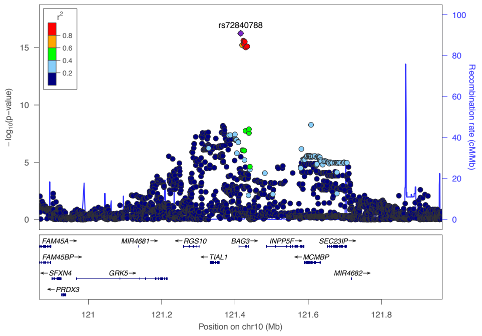

LVEF

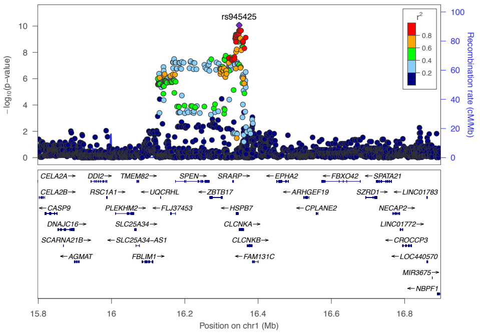

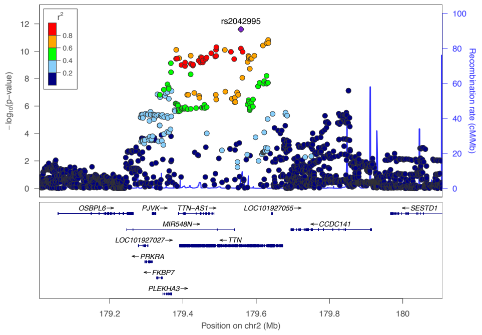

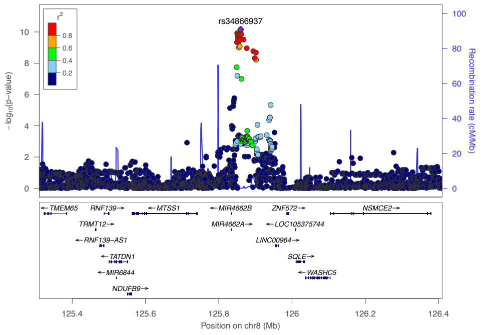

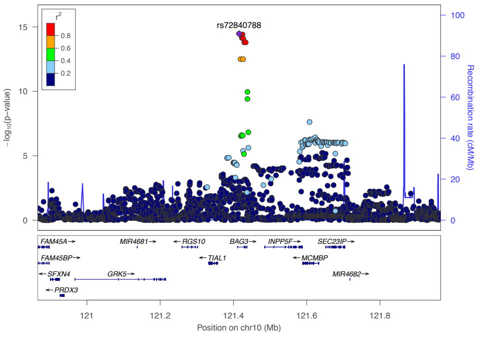

LVM

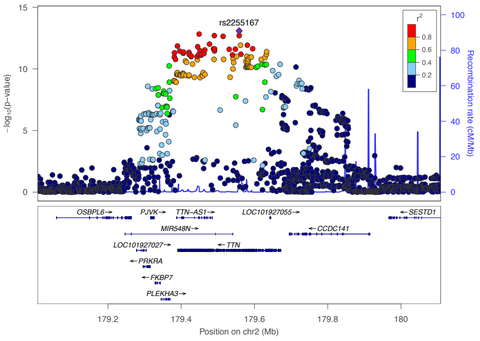

LVMVR

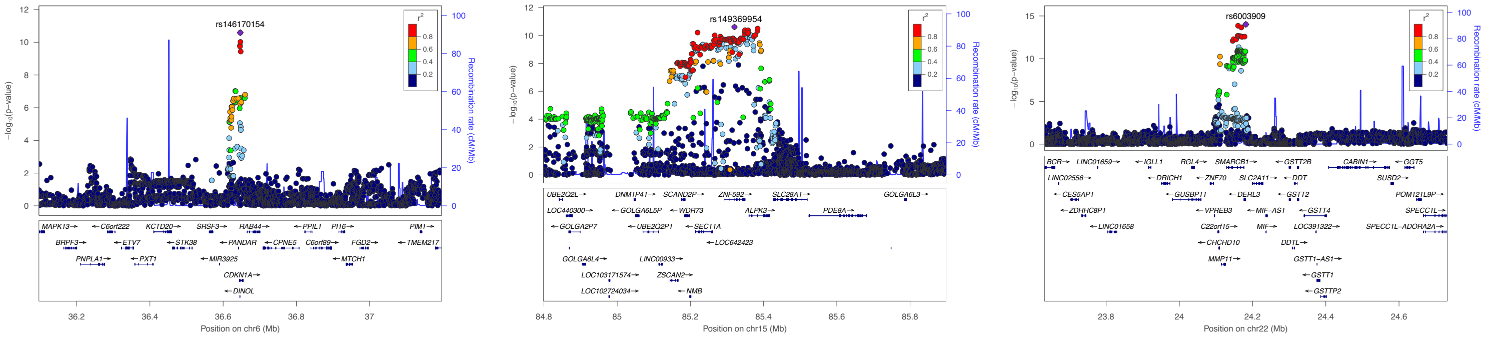

LVEDV, left ventricular end-diastolic volume; LVESV, left ventricular end-systolic volume; LVEF, left ventricular ejection fraction; LVM, left ventricular mass; LVMVR, left ventricular mass to end-diastolic volume ratio

Supplemental Figure 8. Location of the sentinel variants and their proxies in LD $r^2 \geq 0.8$

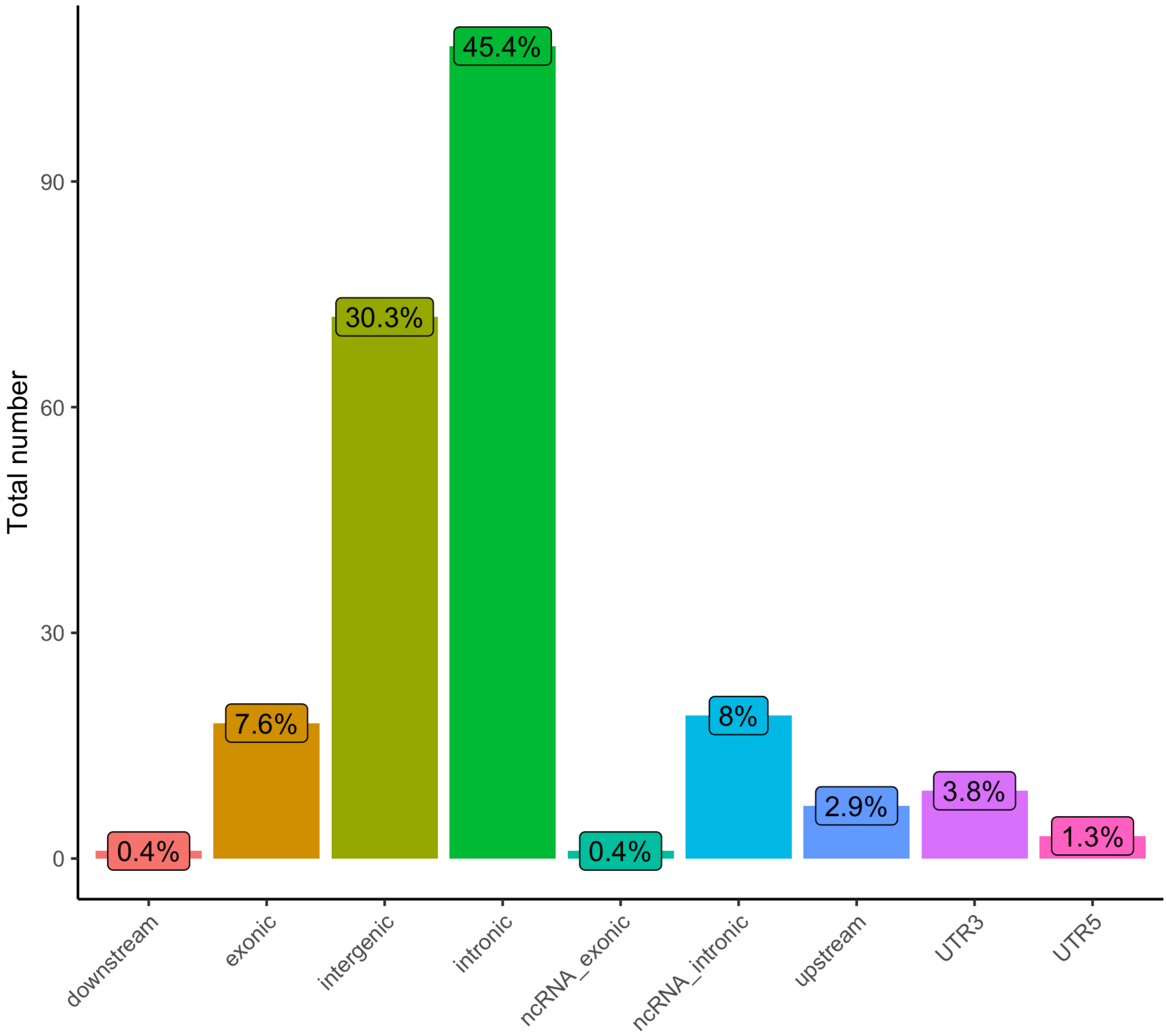

LD, linkage disequilibrium

Supplemental Figure 9. FORGE enrichment analysis

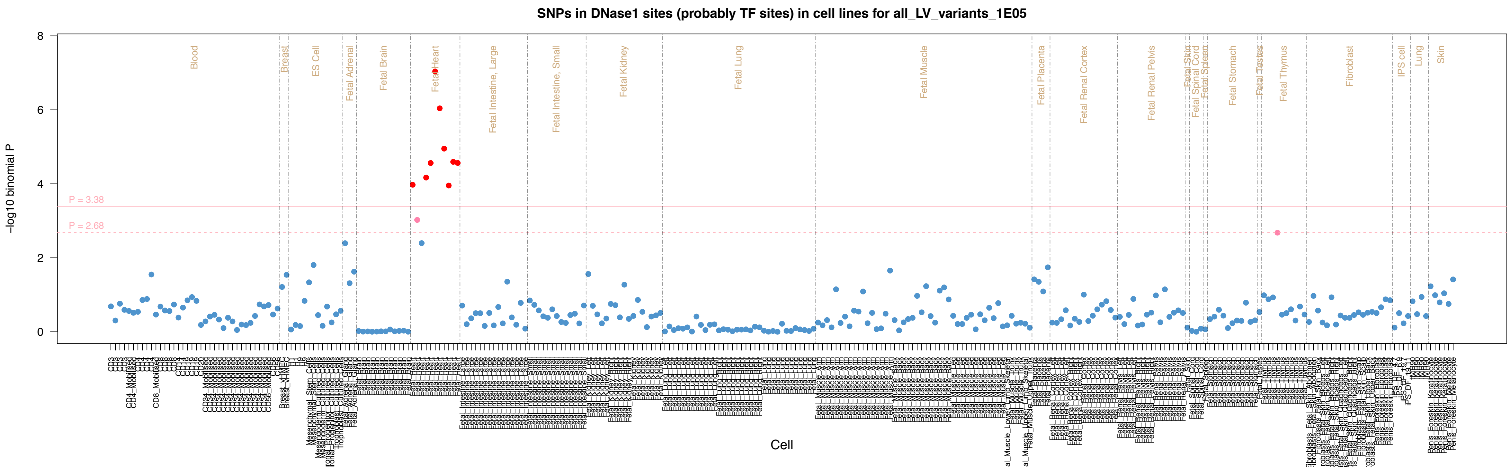

Enrichment of variants associated with LV phenotypes ($P < 1x10^{-5}$) was observed in the Roadmap DNase 1 regulatory regions of fetal heart. The x-axis represents the cell types within different tissues of the Roadmap epigenomics project. The dashed and solid pink lines represent the Bonferroni-corrected p values of 0.05 and 0.01 respectively.

Supplemental Figure 10. Cumulative incidence of heart failure stratified by LV-PRS quintiles

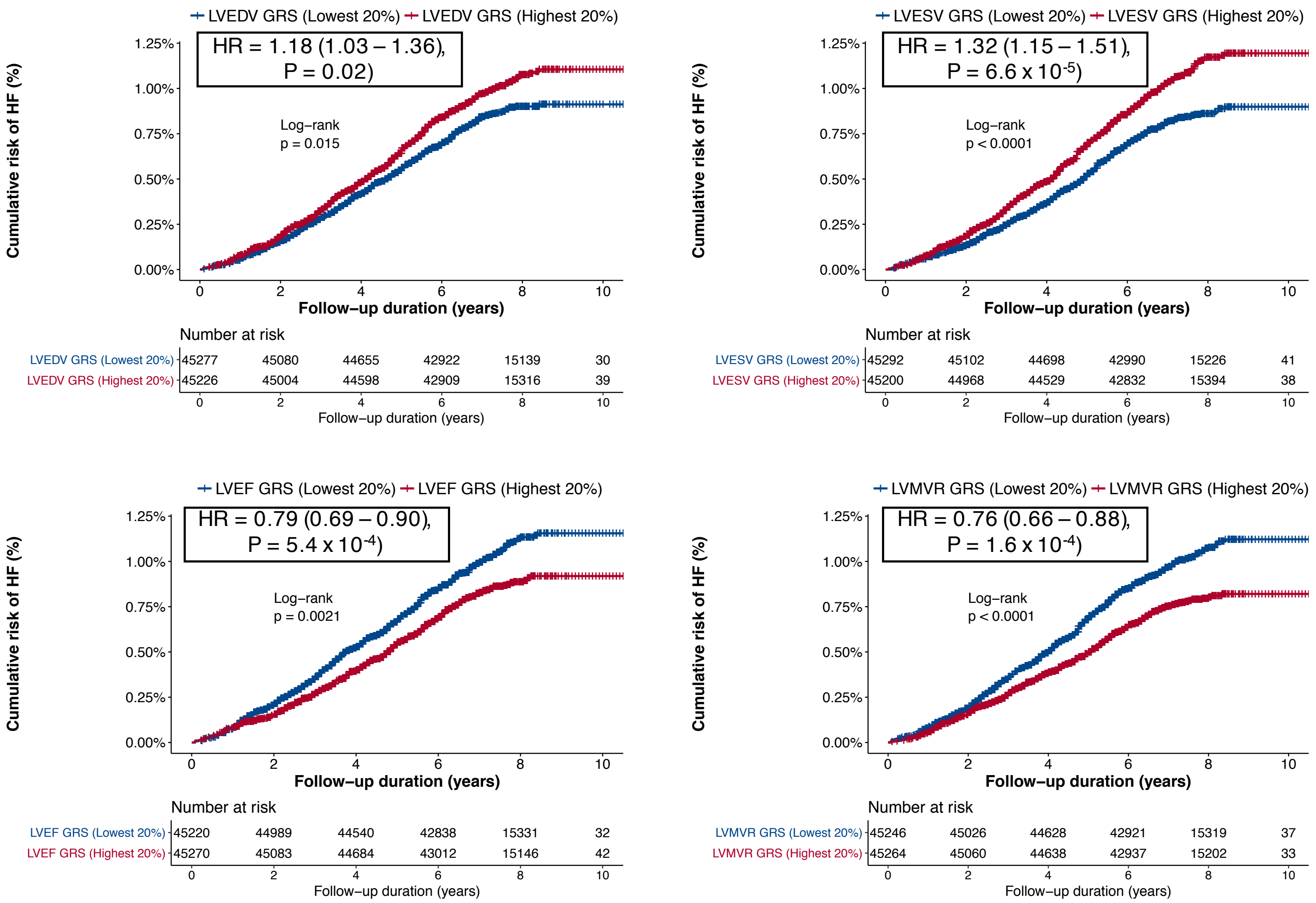

The hazard ratios compared PRS quintile 5 (Highest 20%) vs quintile 1 (Lowest 20%) and were obtained from the multivariate Cox regression models adjusted for age, sex, BMI, SBP and DBP corrected for anti-hypertensive medication use, smoking status, regular alcohol use, dyslipidaemia, diabetes and 15 PCs. There was no significance difference in the incidence of heart failure between the top and bottom quintiles of LVM-PRS (HR = 0.99 [0.87 – 1.13], P = 0.87).

LVEDV, left ventricular end-diastolic volume; LVESV, left ventricular end-systolic volume; LVEF, left ventricular ejection fraction; LVM, left ventricular mass; LVMVR, left ventricular mass to end-diastolic volume ratio; PRS, polygenic risk score; HR, hazard ratio

Supplemental table titles and legends

Supplementary Table 1. Cohort characteristics of the UK Biobank

Numbers are mean (standard deviation) or total number (%). *Systolic and diastolic blood pressure are adjusted for anti-hypertensive medication use by adding 15mmHg and 10mmHg, respectively. CMR, cardiovascular magnetic resonance; LVEDV, left ventricular end-diastolic volume; LVESV, left ventricular end-systolic volume; LVSV, left ventricular stroke volume; LVEF, left ventricular ejection fraction; LVM, left ventricular mass; LVMVR, left ventricular mass to end-diastolic volume ratio

Supplementary Table 2. Conditional analysis in GCTA

Pre-specified criteria for secondary signal: (i) the original p value of newly identified variant was lower than 1×10^{-6}; (ii) there was less than a 1.5-fold difference between the lead SNP and secondary association p values on a –log10 scale, i.e., if –log10(p lead)/-log10(p sec) < 1.5; and (iii) if there was less than a 1.5-fold difference between the main association and conditional association p values on a –log10 scale, i.e., if –log10(p sec)/-log10(p cond) < 1.5. GCTA, genome-wide complex trait analysis tool; UKBB, UK Biobank; LVEDV, left ventricular end-diastolic volume; LVESV, left ventricular end-systolic volume; LVEF, left ventricular ejection fraction; LVM, left ventricular mass; LVMVR, left ventricular mass to end-diastolic volume ratio; CHR, chromosome; EA, effect allele; EAF, effect allele frequency; SE, standard error

Supplementary Table 3. Comparison between primary analysis and secondary analysis with additional adjustment for other cardiovascular risk factors

Secondary analyses were additionally controlled for diastolic blood pressure adjusted for anti-hypertensive medication use, body mass index (instead of weight in the primary model), smoking status, regular alcohol use, dyslipidaemia and diabetes. LVEDV, left ventricular end-diastolic volume; LVESV, left ventricular end-systolic volume; LVEF, left ventricular ejection fraction; LVM, left ventricular mass; LVMVR, left ventricular mass to end-diastolic volume ratio; CHR, chromosome; EA, effect allele; NEA, non-effect allele; EAF, effect allele frequency; SE, standard error

Supplementary Table 4. Comparison between primary analysis and analysis with untransformed LV phenotypes

LVEDV, left ventricular end-diastolic volume; LVESV, left ventricular end-systolic volume; LVEF, left ventricular ejection fraction; LVM, left ventricular mass; LVMVR, left ventricular mass to end-diastolic volume ratio; CHR, chromosome; EA, effect allele; NEA, non-effect allele; EAF, effect allele frequency; SE, standard error

Supplementary Table 5. Interaction analysis of lead variants and systolic blood pressure

Systolic blood pressure was centered and scaled to standard deviation. LVEDV, left ventricular end-diastolic volume; LVESV, left ventricular end-systolic volume; LVEF, left ventricular ejection fraction; LVM, left ventricular mass; LVMVR, left ventricular mass to end-diastolic volume ratio; CHR, chromosome; EA, effect allele; NEA, non-effect allele;

EAF, effect allelic frequency; SE, standard error; SNP, single nucleotide polymorphism; SBP, systolic blood pressure

Supplementary Table 6. Interaction analysis of lead variants and sex

The reference sex was female. The bonferroni significant interaction effects are highlighted in green. LVEDV, left ventricular end-diastolic volume; LVESV, left ventricular end-systolic volume; LVEF, left ventricular ejection fraction; LVM, left ventricular mass; LVMVR, left ventricular mass to end-diastolic volume ratio; CHR, chromosome; EA, effect allele; NEA, non-effect allele; EAF, effect allele frequency; SE, standard error; SNP, single nucleotide polymorphism

Supplementary Table 7. Cohort characteristics of Multi-ethnic Study of Atherosclerosis (MESA)

Numbers are mean (standard deviation) or total number (%). SBP, systolic blood pressure; LVEDV, left ventricular end-diastolic volume; LVESV, left ventricular end-systolic volume; LVSV, left ventricular stroke volume; LVEF, left ventricular ejection fraction; LVM, left ventricular mass; LVMVR, left ventricular mass to end-diastolic volume ratio

Supplementary Table 8. Lookup of the genome-wide significant UK Biobank variants in Multi-ethnic Study of Atherosclerosis (MESA)

Bonferoni-signfiicant variants ($P < 0.0036$ [0.05/14 for multiple testing of 14 variant-trait pairs]) are highlighted in green and nominally-significant variants ($P < 0.05$ with concordant direction of effect) are highlighted in yellow. UKBB, UK Biobank; LVEDV, left ventricular end-diastolic volume; LVESV, left ventricular end-systolic volume; LVEF, left ventricular ejection fraction; LVM, left ventricular mass; LVMVR, left ventricular mass to end-diastolic volume ratio; CHR, chromosome; EA, effect allele; NEA, non-effect allele; EAF, effect allele frequency; SE standard error

Supplementary Table 9. Lookup of previously reported variants for ECHO traits in the UK Biobank

Variants reaching the conventional genome-wide significance level ($P < 5E\text{-}08$) are highlighted in green and nominally-significant variants ($P < 0.05$ with concordant direction of effect) are highlighted in yellow. [*]SH2B3 locus; [†]MTSS1 locus; UKBB, UK Biobank; LVEDV, left ventricular end-diastolic diameter, LVEDD; left ventricular end-systolic diameter, LVESD; left ventricular end-diastolic volume; LVESV, left ventricular end-systolic volume; LVEF, left ventricular ejection fraction; LVM, left ventricular mass; PMID, PubMed ID; CHR, chromosome; EA, effect allele; NEA, non-effect allele; EAF, effect allele frequency; SE, standard error

Supplementary Table 10. Lookup of previously reported variants for LVH identified by ECG in the UK Biobank LVM GWAS

Nominally-significant variants ($P < 0.05$ with concordant direction of effect) are highlighted in yellow. LVM, left ventricular mass; ECG-LVH, left ventricular hypertrophy identified by electrocardiogram; CHR, chromosome; EA, effect allele; NEA, non-effect allele; EAF, effect allele frequency; SE, standard error

Supplementary Table 11. Lookup of the UK Biobank sentinel variants and their proxies (LD r2 >= 0.8) in Phenoscanner v2

Only associations at $p < 5x10^{-8}$ are shown in the table. Variants associated with relevant cardiovascular traits/diseases are highlighted in green. LD, linkage disequilibrium; UKBB, UK Biobank; CMR, cardiovascular magnetic resonance; LVEDV, left ventricular end-diastolic volume; LVESV, left ventricular end-systolic volume; LVEF, left ventricular ejection fraction; LVMVR, left ventricular mass to end-diastolic volume ratio; EA, effect allele; NEA, non-effect allele; SE, standard error; OR, odds ratio; IVNT, rank-based inverse normal transformation

Supplementary Table 12. Lookup of the UK Biobank sentinel variants and their proxies (LD r2 >= 0.8) in Gene Atlas dataset

Only associations at $p < 5x10^{-8}$ are shown in the table. Variants associated with relevant cardiovascular traits/diseases are highlighted in green. UKBB, UK Biobank; LD, linkage disequilibrium; CMR, cardiovascular magnetic resonance; LVEDV, left ventricular end-diastolic volume; LVESV, left ventricular end-systolic volume; LVEF, left ventricular ejection fraction; SNP, single nucleotide polymorphism; EA, effect allele; OR, odds ratio; MAF, minor allele frequency

Supplementary Table 13. Lookup of the UK Biobank sentinel variants and their proxies (LD r2 >= 0.8) in GTEx dataset

Variants significantly associated with gene expression in cardiovascular tissue (at FDR < 0.05) are highlighted in green. FDR, false discovery rate; UKBB, UK Biobank; LD, linkage disequilibrium; CMR, cardiovascular magnetic resonance; LVEDV, left ventricular end-diastolic volume; LVESV, left ventricular end-systolic volume; LVEF, left ventricular ejection fraction; LVM, left ventricular mass; LVMVR, left ventricular mass to end-diastolic volume ratio

Supplementary Table 14. Long-range chromatin interaction analysis

[*]Lead variant for LVM is rs2255167. UKBB, UK biobank; CMR, cardiovascular magnetic resonance; LVEDV, left ventricular end-diastolic volume; LVESV, left ventricular end-systolic volume; LVEF, left ventricular ejection fraction; LVM, left ventricular mass; LVMVR, left ventricular mass to end-diastolic volume ratio; CHR, chromosome

Supplementary Table 15. Summary of variant-level annotations

[*]Lead variant in TTN locus for LVM; [†]Lead variant in TTN locus for LVEDV, LVESV, LVEF; [‡]Lead variant in MTSS1 locus for LVEF; [§]Lead variant in MTSS1 locus for LVESV. CMR, cardiovascular magnetic resonance; LVEDV, left ventricular end-diastolic volume; LVESV, left ventricular end-systolic volume; LVEF, left ventricular ejection fraction; LVM, left ventricular mass; LVMVR, left ventricular mass to end-diastolic volume ratio; CHR, chromosome; EA, effect allele; NEA, non-effect allele; EAF, effect allele frequency; AA, amino acid

Supplementary Table 16. Summary of gene-level annotations

[*]Lead variant for LVEDV is rs7071853. [†]Lead variant for LVEF is rs34866937. [‡]Lead variant for LVM is rs2255167. UKBB, UK Biobank; CMR, cardiovascular magnetic resonance; LVEDV, left ventricular end-diastolic volume; LVESV, left ventricular end-systolic volume; LVEF, left ventricular ejection fraction; LVM, left ventricular mass; LVMVR, left ventricular mass to end-diastolic volume ratio; CHR, chromosome; CV, cardiovascular

Supplementary Table 17. GeneNetwork pathway analyses

GO, gene ontology

Supplemental References

1. Bai W, Sinclair M, Tarroni G, Oktay O, Rajchl M, Vaillant G, Lee AM, Aung N, Lukaschuk E, Sanghvi MM, et al. Automated cardiovascular magnetic resonance image analysis with fully convolutional networks. *J Cardiovasc Magn Reson*. 2018;20:65.

2. R Core Team. R: A Language and Environment for Statistical Computing [Internet]. Vienna, Austria: R Foundation for Statistical Computing; 2016. Available from: https://www.R-project.org/

3. Yang J, Ferreira T, Morris AP, Medland SE, Madden PAF, Heath AC, Martin NG, Montgomery GW, Weedon MN, Loos RJ, et al. Conditional and joint multiple-SNP analysis of GWAS summary statistics identifies additional variants influencing complex traits. *Nat Genet*. 2012;44:369-S3.

4. Bild DE, Bluemke DA, Burke GL, Detrano R, Diez Roux AV, Folsom AR, Greenland P, JacobsJr. DR, Kronmal R, Liu K, et al. Multi-Ethnic Study of Atherosclerosis: Objectives and Design. *Am J Epidemiol*. 2002;156:871–881.

5. Tobin MD, Sheehan NA, Scurrah KJ, Burton PR. Adjusting for treatment effects in studies of quantitative traits: antihypertensive therapy and systolic blood pressure. *Stat Med*. 2005;24:2911–2935.

6. Manichaikul A, Naj AC, Herrington D, Post W, Rich SS, Rodriguez A. Association of SCARB1 variants with subclinical atherosclerosis and incident cardiovascular disease: the multi-ethnic study of atherosclerosis. *Arterioscler Thromb Vasc Biol*. 2012;32:1991–1999.

7. Patterson N, Price AL, Reich D. Population Structure and Eigenanalysis. *PLOS Genet*. 2006;2:e190.

8. Price AL, Patterson NJ, Plenge RM, Weinblatt ME, Shadick NA, Reich D. Principal components analysis corrects for stratification in genome-wide association studies. *Nat Genet*. 2006;38:904–909.

9. Aulchenko YS, Struchalin MV, van Duijn CM. ProbABEL package for genome-wide association analysis of imputed data. *BMC Bioinformatics*. 2010;11:134.

10. Wang K, Li M, Hakonarson H. ANNOVAR: functional annotation of genetic variants from high-throughput sequencing data. *Nucleic Acids Res*. 2010;38:e164.

11. Ward LD, Kellis M. HaploReg: a resource for exploring chromatin states, conservation, and regulatory motif alterations within sets of genetically linked variants. *Nucleic Acids Res*. 2012;40:D930–D934.

12. Boyle AP, Hong EL, Hariharan M, Cheng Y, Schaub MA, Kasowski M, Karczewski KJ, Park J, Hitz BC, Weng S, et al. Annotation of functional variation in personal genomes using RegulomeDB. *Genome Res*. 2012;22:1790–1797.

13. Lonsdale J, Thomas J, Salvatore M, Phillips R, Lo E, Shad S, Hasz R, Walters G, Garcia F, Young N, et al. The Genotype-Tissue Expression (GTEx) project. *Nat Genet*. 2013;45:580–585.

14. Watanabe K, Taskesen E, Bochoven A, Posthuma D. Functional mapping and annotation of genetic associations with FUMA. *Nat Commun*. 2017;8:1826.

15. Schmitt AD, Hu M, Jung I, Xu Z, Qiu Y, Tan CL, Li Y, Lin S, Lin Y, Barr CL, et al. A Compendium of Chromatin Contact Maps Reveal Spatially Active Regions in the Human Genome. *Cell Rep*. 2016;17:2042–2059.

16. Pers TH, Karjalainen JM, Chan Y, Westra H-J, Wood AR, Yang J, Lui JC, Vedantam S, Gustafsson S, Esko T, et al. Biological interpretation of genome-wide association studies using predicted gene functions. *Nat Commun*. 2015;6:5890.

17. Dunham I, Kulesha E, Iotchkova V, Morganella S, Birney E. FORGE: A tool to discover cell specific enrichments of GWAS associated SNPs in regulatory regions. *F1000Research*. 2015;4:18.

18. Quinlan AR, Hall IM. BEDTools: a flexible suite of utilities for comparing genomic features. *Bioinformatics*. 2010;26:841–842.

19. Deelen P, Dam S van, Herkert JC, Karjalainen JM, Brugge H, Abbott KM, Diemen CC van, Zwaag PA van der, Gerkes EH, Folkertsma P, et al. Improving the diagnostic yield of exome-sequencing, by predicting gene-phenotype associations using large-scale gene expression analysis. *bioRxiv*. 2018;375766.

20. Stelzer G, Rosen N, Plaschkes I, Zimmerman S, Twik M, Fishilevich S, Stein TI, Nudel R, Lieder I, Mazor Y, et al. The GeneCards Suite: From Gene Data Mining to Disease Genome Sequence Analyses. *Curr Protoc Bioinforma*. 2016;54:1.30.1-1.30.33.

21. Vilhjálmsson BJ, Yang J, Finucane HK, Gusev A, Lindström S, Ripke S, Genovese G, Loh P-R, Bhatia G, Do R, et al. Modeling Linkage Disequilibrium Increases Accuracy of Polygenic Risk Scores. *Am J Hum Genet*. 2015;97:576–592.
